# Toward Gaussian Process Regression Modeling of a Urea Force Field

**DOI:** 10.1021/acs.jpca.4c04117

**Published:** 2024-09-20

**Authors:** Tomasz Bukowy, Matthew L. Brown, Paul L. A. Popelier

**Affiliations:** Department of Chemistry, University of Manchester, Manchester M13 9PL, Great Britain

## Abstract

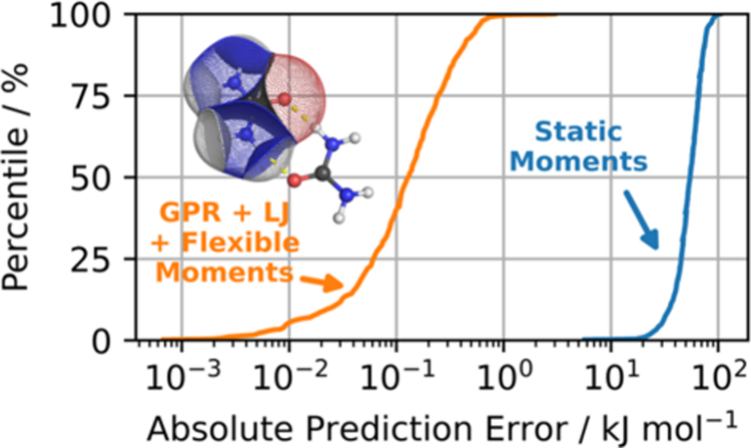

FFLUX is a next-generation, machine-learnt force field built on three cornerstones: quantum chemical topology, Gaussian process regression, and (high-rank) multipolar electrostatics. It is capable of performing molecular dynamics with near-quantum accuracy at a lower computational cost than standard *ab initio* molecular dynamics. Previous work with FFLUX was concerned with water and formamide. In this study, we go one step further and challenge FFLUX to model urea, a larger and more flexible system. In result, we have trained urea models at the B3LYP/aug-cc-pVTZ level of theory, with a mean absolute error of 0.4 kJ mol^–1^ and a maximum prediction error below 7.0 kJ mol^–1^. To test their performance in molecular dynamics simulations, two sets of FFLUX geometry optimizations were carried out: 5 dimers corresponding to energy minima and 75 random dimers. The 5 dimers were recovered with a root-mean-square deviation below 0.1 Å with respect to their *ab initio* references. Out of the 75 random dimers, 68% converged to the qualitatively same dimer as those obtained at the *ab initio* level. Furthermore, we have ranked the 5 FFLUX-optimized dimers in the order of their relative FFLUX single-point energies and compared them with the *ab initio* method. The energy ranking fully agreed but for one crossover between two successive minima. Finally, we have demonstrated the importance of geometry-dependent (*i*.*e*., flexible) multipole moments, showing that the lack of multipole moment flexibility can lead to average errors in the total intermolecular electrostatic energy of more than 2 orders of magnitude.

## 1 Introduction

One of the most common tools used to rationalize an experimental outcome is the classical force field, which allows for the modeling of large chemical systems at time scales inaccessible to *ab initio* methods. The price paid for this advantage is that force fields risk sacrificing accuracy.^1,2^ This limitation mainly arises from the facile description of electrostatics, *i*.*e*., the use of mere static point charges (“a monopoly of monopoles”), which disregards the spatial, nonspherical complexity of electron density.^3^ Over the years, it has been increasingly recognized that classical force fields need to be equipped with multipole moments to make more reliable predictions. Some examples include AMOEBA+,^4,5^ SIBFA21,^6^ DIFF,^7^ MASTIFF,^8^ Slater-FF^9^ and GEM.^10^

However, problems with classical force fields run deeper than the well-known shortcoming of point charges. Typical force field design is inherently flawed because there is a nonphysical inconsistency between their bonded and nonbonded potentials. Indeed, first, only the latter comprises the Lennard-Jones potential, which contains interatomic dispersion. Yet, one can argue that this type of interaction, as a product of electron correlation, is actually present between any pair of atoms, regardless of their bonded or nonbonded status.^11^ Second, electrostatics is not represented between bonded atoms, or even within an atom. Yet, it is indeed present, physically. Instead, point charges that represent electrostatics appear only from 1–4 interactions onward, curiously. To top it all, the parametrization with respect to bonds, valence angles, and dihedral angles poses a source of significant transferability issues.

DL_FFLUX^12,13^ is the code that houses the FFLUX force field or rather a collection of machine-learnt potentials as FFLUX can be perceived in its current state of development. FFLUX aims to obtain the best of both worlds: the accuracy of *ab initio* calculations and the speed of a classical force field, overcoming the shortcomings of the latter with a novel design. This *ab ovo* approach involves the machine learning method Gaussian process regression^14^ (GPR). FFLUX is GPR-trained on atomic energies generated by the interacting quantum atoms (IQA) energy partitioning scheme. Moreover, FFLUX also has access to trained atomic multipole moments, again obtained in the context of quantum topological atoms, which also underpin IQA. This uniformity in the way both atomic multipole moments and energies are generated by the same quantum topological partitioning is a strength of this approach. The multipole moments represent the long-range energetics, which is thus seamlessly connected to the aforementioned atomic energies, which are short-range by nature.

FFLUX allows simulations of fully flexible molecules with flexible multipole moments. This capability was impossible for decades in the context of an early, well-known multipolar scheme called Distributed Multipole Analysis (DMA), *i*.*e*., multipole moments that are explicitly dependent on atomic positions, and can change during the runtime of a molecular dynamics (MD) simulation. While this capacity has previously been achieved with the AMOEBA+CF force field,^5^ where geometry-dependent charges were implemented, in FFLUX *all* multipole moments (including charge) up to the hexadecapole moment are geometry-dependent. The GPR models can be trained for any chemical system and have a good track record regarding their accuracy^15,16^ and transferability.^17^ The FFLUX force field has previously been used to successfully calculate various properties of liquid water^18^ as well as the geometries of formamide dimers^19^ and crystals.^20^ Moreover, phonon calculations were found to be possible using FFLUX, thereby allowing access to Helmholtz free energies, with calculations being 10^5^ times faster than DFT+D calculations.

**Figure 1:**
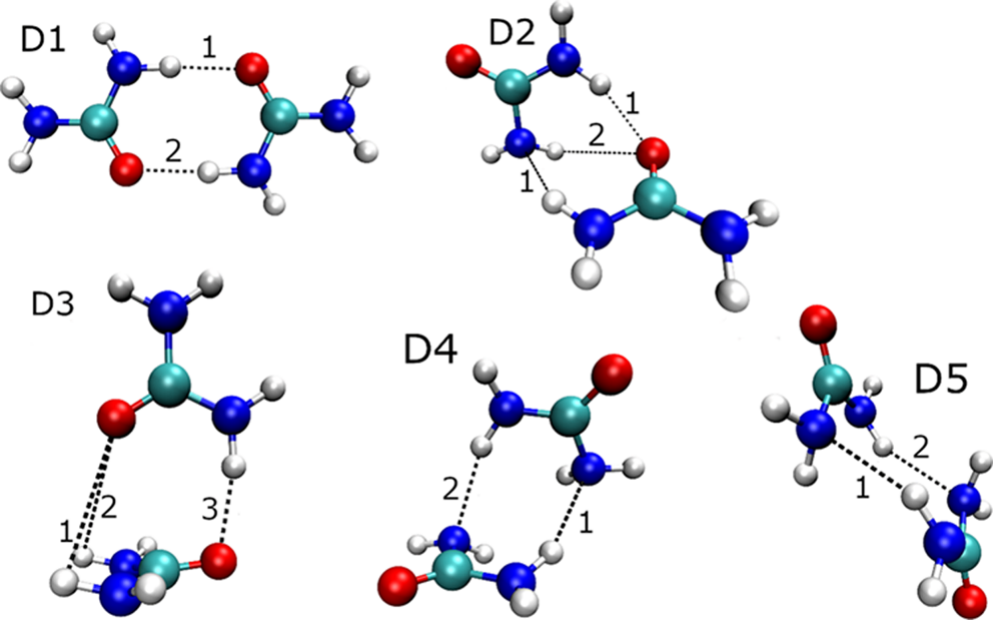
Previously identified^23^ urea dimers and reoptimized at the B3LYP/aug-cc-pVTZ-D3 level of theory for this work’s sake. Numbering of hydrogen bonds corresponds with the numbering in Table 2.

Urea is a highly studied compound in chemistry owing to its importance and ubiquity in nature. The presence of two nitrogen and one oxygen atoms in its structure allows it to readily form hydrogen bonding, and therefore a variety of complex clusters.^21,22^ This makes urea an apt system to test the methodology behind FFLUX with, as multipolar electrostatics are essential to capture directionality, the hallmark of a hydrogen bond. The urea monomer and dimers have been extensively researched computationally over the years.^21,23,24^ One of the most comprehensive studies^23^ confirmed the global minimum, *D1*, and the local minima *D2*, *D3*, *D4*, *D5* (shown in Figure 1) at a selection of *ab initio* and semiempirical levels of theory: HF, MP2, DFT-B3PW91, AM1, PM3 and SAM1. In their work, the optimized structures varied quantitatively but their relative energies were generally unchanged across the different methods. For the current work, we reoptimized these dimers at the B3LYP-D3/aug-cc-pVTZ level of theory and labeled them according to increasing energy. The monomer as well as the above-mentioned equilibrium geometry dimers have been used as test systems for the performance of the predicted IQA energies, multipolar electrostatics and the chosen nonbonded potential. The exact coordinates of the dimers are available in Section 1 of the Supporting Information (SI).

In this work, we benchmark the performance of our GPR models in urea monomers and dimers. For the former we use monomeric geometries randomly sampled from a classical MD trajectory, and compared true and predicted atomic IQA energies. We also recover the single-point energy and the dipole moment of the global minimum. For the latter, we test our multipole moment models on dimeric geometries randomly sampled from an *ab initio* MD trajectory. With an additional van der Waals potential, we then optimize five dimers using FFLUX and compare their geometries as well as single-point energies against B3LYP-D3/aug-cc-pVTZ results. Finally, we compare the optimization convergence of randomly arranged dimers between FFLUX and B3LYP-D3/aug-cc-pVTZ, and make a case for the use of flexible multipole moments in MD.

## 2 Methods

The FFLUX force field is implemented as an add-on to an open-source MD software, DL_POLY_4,^25^ as DL_FFLUX. The methodology behind FFLUX has been laid out exhaustively in previous publications.^12,13^ However, some essential ideas behind FFLUX are briefly described in this section.

### 2.1 Quantum Theory of Atoms in Molecules

The first cornerstone of FFLUX is the Quantum Theory of Atoms in Molecules (QTAIM),^26^ which is a topological partitioning of molecular charge density into space-filling regions, or topological atoms. Boundaries between such atoms are interatomic surface (IAS) satisfying eq 1
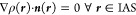
1

**Figure 2:**
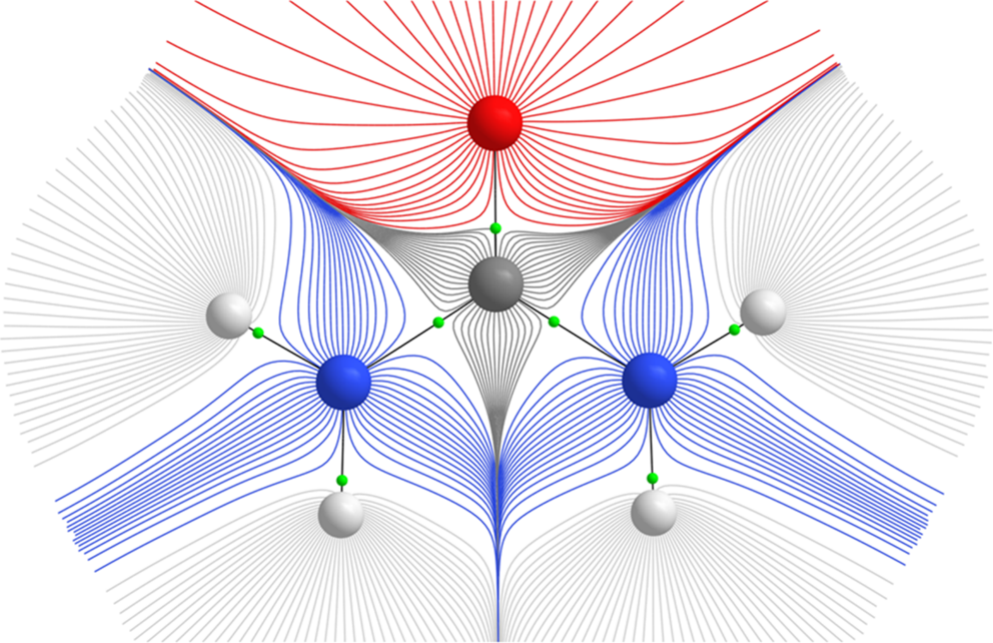
Topological partitioning of the electron density of urea indicated by gradient paths with bond critical points (green).

If point ***r*** belongs to an IAS, then the vector normal to it, ***n***(***r***), is orthogonal to the gradient path (*i*.*e*., path of the steepest ascent), which can be seen as sequence of very short gradient vectors, ∇ρ(***r***). QTAIM requires no corrections for penetration energies, nor reference density, as the gradient paths are traced out in a nonoverlapping manner, which is illustrated in Figure 2 by the example of urea. The minima in the direction of neighboring nuclei are referred to as bond critical points (BCPs). Together with IASs surfaces they form Morse-Smale complexes of electron density.

### 2.2 Interacting Quantum Atoms

Over the decades, the key idea behind QTAIM was adopted in the study of 3D quantum mechanical functions other than the electron density, leading to a set of methods called Quantum Chemical Topology (QCT). A now much used method among them, called Interacting Quantum Atoms (IQA),^27^ enables, unlike QTAIM, the energy partitioning of *nonequilibrium* geometries into atomic energies using one- and two-electron density matrices
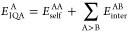
2
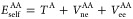
3
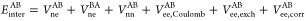
4An IQA atomic energy, *E*_IQA_^A^, comprises two types of energy: intra-atomic energy, *E*_self_^AA^, and interatomic energy, *E*_inter_^AB^. These contributions can be further broken down: *E*_self_^AA^ consists of the electronic kinetic energy, *T*^A^, electron–nucleus attraction (*V*_ne_^AA^) and electron–electron repulsion (*V*_ee_^AA^) energies. Looking at eq 4, *E*_inter_^AB^ has atom-pairwise contributions: the attraction energy between the nucleus of atom A and the electrons of atom B, denoted *V*_ne_^AB^, conversely, the attraction energy between the nucleus of atom B and the electrons of atom *A*, *V*_ne_^BA^, the repulsion energy between nuclei A and B, the Coulomb energy between electrons of A and of B, *V*_ee,Coulomb_^AB^, and exchange and correlation electronic energies, *V*_ee,exch_^AB^, and *V*_ee,corr_^AB^, respectively. The first four terms of eq 4 are orbital-independent in the sense that they can be written using the electron density only. They are classical electrostatic contributions that can be expanded using multipole moments (Section 2.4). The last two energy terms cannot be written in terms of only electron density but explicitly refer to orbitals.

### 2.3 Gaussian Process Regression (GPR)

GPR is a nonparametric Bayesian machine learning method^28^ that is increasingly popular within potential energy surface prediction since the method’s introduction^29^ to this type of research (be it *via* atomic multipole moments) in 2009. GPR is implemented in the in-house engine FEREBUS^30^ that generates models, which in turn are used by FFLUX to make predictions of atomic energies, *E*_IQA_^A^, and IQA-compatible multipole moments from a sample of molecular geometries. The atomic energy models replace the potentials used for bonds, angles and dihedrals in traditional force fields, and enable the recovery of all intramolecular short-range interactions, preserving their inherent many-body nature. Second, the atomic multipole moments, together with a parametric nonbonded potential, handle long-range electrostatics, necessary to model intermolecular interactions.

To train a given model, molecular geometries are passed as input vectors with reference to atomic local frames (ALFs).^31^ An ALF is constructed by placing the atom being trained for (A) at the origin of the coordinate system. Then, the Cahn–Ingold–Prelog rules are used to select two other atoms of highest and second-highest priority, *i*.*e*. A_*x*_ and A_*xy*_, which are needed to respectively fix the *x*-axis and *xy* plane. The *z*-axis is constructed orthogonally to the *xy* plane to form a right-handed axis system. The first (machine learning) feature is the distance between atom A and A_*x*_, the second feature is the distance between atom A and A_*xy*_, and the third feature is the A_*x*_–A–A_*xy*_ angle. The remaining atoms’ positions are encoded as spherical coordinates relative to the ALF.

For any two so-encoded molecular geometries in the training set, GPR uses a radial basis function (RBF) kernel as a variogram that measures the covariance between them. All possible pairs of geometries thus form a covariance matrix, which depends on a set of hyperparameters, θ_*h*_. They are optimized by maximizing the concentrated log-likelihood during training after which they are fed back into the covariance matrix to predict an IQA energy or multipole moment for a previously unseen geometry using eq 5
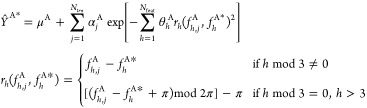
5where *Ŷ*^A^ is the predicted property for atom A, μ^A^ is the mean value of the predicted property within the training set, and α_j_^A^ is the weight of *j*th training point. The quantities *f*_h,j_^A^ and *f*_h,j_^A*^ are vectors containing ALF encoded geometries, for *j*th training and unseen geometries, respectively. Note that the kernel *r_h_(f*_h,j_^A^,*f*_h,j_^A*^) has been modified in order to account for the fact that every third feature is an angle that takes a value from −π to π.

The above scheme is implemented in the in-house Python pipeline called ICHOR,^32^ which automates a few essential pieces of software in the following order:

(1) Generation of molecular geometries using AMBER^33^ or CP2K^34^ MD software.
(2) Calculation of wave function for each molecular geometry using GAUSSIAN09.^35^
(3) Calculation of IQA energy and multipole moments from the wave function using AIMAll.^36^
(4) Optimization of the GPR hyperparameters and creation of model files containing optimized hyperparameters using the FEREBUS^30^ engine.
(5) Addition of new points to the training set by the adaptive sampling (active learning) “mean absolute prediction error” (MEPE) acquisition algorithm^37^ that was implemented in-house.^16^

For the training of the urea models featured in this work, a million geometries were generated from a 1 ns, 500 K AMBER urea monomer simulation and down-sampled (*i*.*e*. reduced in size) by ICHOR to generate three sets of geometries: an initial training set (54 geometries), a validation set (10,000 geometries) and a sample pool (100,000). The initial training set was created based on the minimum, mean, and maximum of each of the 3*N*–6 features in the trajectory. The validation set, and the sample pool were randomly generated, ensuring that both sets were unique, and no single geometry appeared in both sets. From the sample pool the MEPE algorithm selected new points and added them to the training set iteratively in batches, as the training progressed. All atoms in the system were trained “per-atom”, where each trained atom in the system had its own unique training set. All the models used in this work were trained with 2000 geometries.

### 2.4 Multipolar Electrostatics and Ewald Summation

As it solely depends on charge density, the purely electrostatic energy (the first 4 terms in the eq 4) can be multipole-expanded in a Legendre polynomial basis using the tensor interaction formula (eq 6) that calculates in a convergent manner how an infinitesimal piece of charge density interacts with another elsewhere in space^3^
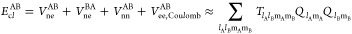
6where *T*_*l*_A_*l*_B_m_A_m_B__ is an interaction tensor keeping track of local coordinates and geometries, and the “approximately equal” sign refers to the practical error introduced by truncating the series expansion. The spherical tensor based (atomic) multipole moments, *Q*_*l*_A_m_A__ and *Q*_*l*_B_m_B__, for atoms A and B, are more compact than their Cartesian counterpart and contain no redundancies. The rank *l* = 0, 1, 2..., determines the type of multipole moment (respectively, monopole, dipole, quadrupole) while *m* = −l,..., 0, ...l determines one of the 2*l* + 1 components. In practice, this infinite sum is truncated at a certain value of *l*. FFLUX uses predicted multipole moments to calculate long-range (intermolecular) electrostatic energy contributions as per eq 6, which has been implemented in house as an extension of the program DL_POLY 4. However, throughout this work we have used our alternative method of the smooth particle mesh Ewald (SPME)^38^ that uses Cartesian moments to account for the periodic boundary conditions. Moreover, both methods have been adapted^39^ in FFLUX such that it allows the computation of *flexible* multipole moments that can change with the geometry of the system.

Short-range (intramolecular) electrostatics are baked in the *Ê*_IQA_^A^ term from which intramolecular forces can be calculated as per eq 7
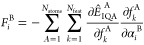
7where *F*_*i*_^B^ is the *i*th force component acting on atom *B*, *f*_*k*_^A^ are the features of atom *A* inducing a force on atom B, while α_*i*_^B^ is atom B’s *i*th coordinate with respect to the global frame. As previously mentioned, such formulation liberates the force field from the bonded potentials used in classical force fields.

## 3 Results and Discussion

### 3.1 Quality of the GPR Urea Model

#### 3.1.1 S-Curves

Establishing the prediction quality of an atomic property requires a validation set of geometries, unknown to the model but derived from the same sample pool as the training set. The prediction error can then be calculated as follows
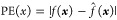
8where *f*(***x***) and *f̂*(***x***) are respectively the true and predicted IQA property values, and ***x*** is a feature vector. A holistic way to monitor the prediction error within a GPR validation set is the cumulative error distribution, which we have been calling S-curves because of their typical sigmoidal shape. An S-curve plots the percentile expressing the fraction (*y*-axis) of validation set geometries that have a prediction error smaller than a given value on the *x*-axis (typically logarithmic).

**Figure 3:**
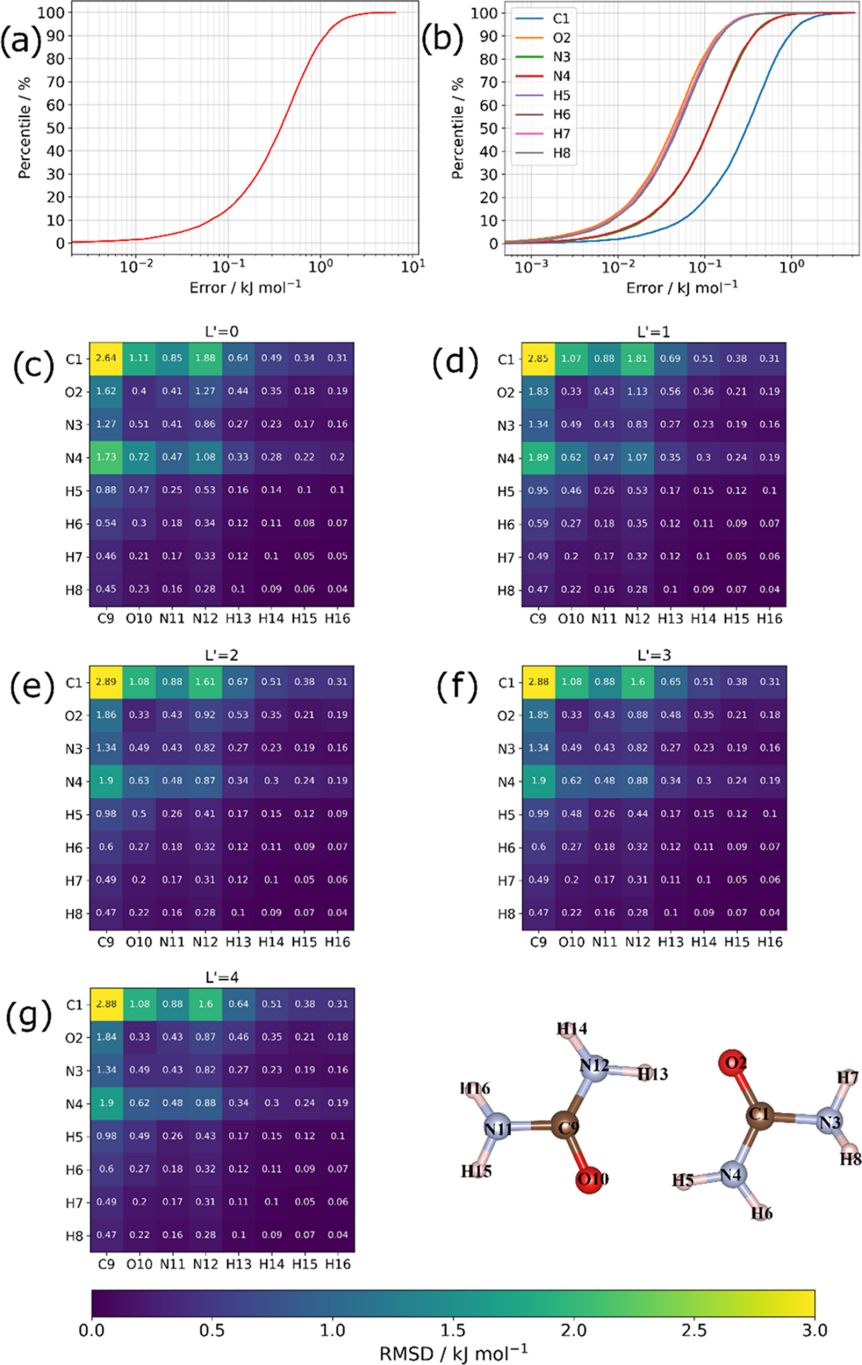
Validation of the 2000-point urea GPR model. (a) S-curve showing the error in the predicted IQA total energies for each geometry in a 10,000-point training set. (b) S-curves showing the error in the predicted IQA energies for each atom. In both (a) and (b), the prediction errors are plotted against percentile. (c–g) Root-mean-squared error (RMSE) in the predicted multipole moments converted to electrostatic energies for intermolecular atom–atom interactions across a 500-point validation set of urea dimers generated with a 300 K CP2K simulation. Electrostatic energies were calculated using truncations of the multipole expansion determined by the value *L*′, which indicates the highest rank present in the simulation. For example, an *L*′ value of 0 indicates monopoles (charges) interacting with monopoles only, whereas a value of 4 indicates that all possible interactions up to the hexadecapole moment are used, and (c) corresponds to *L*′ = 0; (d) to *L*′ = 1, ...; and (g) to *L*′ = 4.

The S-curves in Figure 3a shows prediction errors in atomic IQA energies for the total system and Figure 3b for the individual atoms where a validation set of 10,000 monomeric geometries was used. This large number of geometries makes the S-curves very smooth and justifies their precise read-off. In other words, if another validation set of 10,000 had been chosen the S-curves would have looked the same, which cannot be said for a validation with only a few hundred geometries. The total system S-curve passes the 50th percentile at 0.4 kJ mol^–1^, showing that half the total IQA energy errors fall well within the often-quoted value for chemical accuracy (1 kcal mol^–1^ or 4.2 kJ mol^–1^),^40^ in fact, an order of magnitude smaller. The errors of carbon contribute the most because its S-curve lies the most to the right in Figure 3b. These high errors are due to carbon’s location among the three electronegative atoms (*i*.*e*. two nitrogens and one oxygen), which causes high variability of electron density in its atomic basin across geometries. The maximum total IQA error is nearly 7.0 kJ mol^–1^. However, we do not attach as much importance to this metric because it is highly dependent on the size of the validation set and the occurrence of outliers as shown in Figure S7 of Section 2 of the SI.

The assessment of the quality of the electrostatic multipole moment predictions is slightly more involved due to the “raw” atomic multipole moments being predicted in the units of *e Bohr*^*l*^, where *l* is the rank of the moment. Section 2.2 of the SI shows all the S-curves (Figures S8–S32), that is, for each of the 25 multipole moments of each atom. In general, the trends therein mirror that of the IQA energy: hydrogens perform the best, followed by oxygen, nitrogens, and finally carbon. It is more convenient to assess the predictions of these multipole moments collectively by invoking the right-hand side of eq 6. The idea is to let all predicted multipole moments of one monomer interact with all predicted multipole moments of the other in a pairwise manner, and then let the same happen for the true moments. This gives two interaction energy values (predicted and true) in the more familiar kJ mol^–1^, rather than dispersed in units of *e* Bohr^*l*^, the difference between which is the interaction error for a given *L*′. The parameter *L*′ refers to the presence of all possible multipole moment interactions of rank up to, and including, *L*′ in a given calculation^12^ where *L*′ = 0 denotes the monopole moment (*i*.*e*., charge), and *L*′ = 4 the hexadecapole. For instance, the simplest interaction error conversion from moment to energy is that for atomic charges, which can be expressed^17^ using eq 9
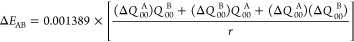
9where Δ*Q*_00_^A^ and Δ*Q*_00_^B^ are the differences between the predicted and true values of charges A and B, and *Q*_00_^A^ and *Q*_00_^B^ are the true charges of A and B (both expressed in milli-electrons abbreviated *me*) while *r* is internuclear distance in Å. The prefactor accounts for unit conversions allowing an energy error to be recovered in kJ mol^–1^. For example, atoms with charge errors of 1 me and true charges of 1500 me (both) at a distance of 2.0 Å away will generate an error of about 2 kJ mol^–1^.

#### 3.1.2 Assessment of Multipole Moments by Dimeric Electrostatic Energies

For the purpose of such a dimeric test of electrostatics, a 300 K CP2K simulation was run at the B3LYP/6-31G* level of theory with dimer *D1* as the starting geometry. A set of 500 geometries was randomly sampled from the trajectory to form the dimeric validation set. Then, using DL_FFLUX in validation mode, intermolecular atom-pairwise electrostatic energies were calculated from predicted multipole moments for *L*′ = 0,1,2,3,4, and compared with the energies calculated using the multipole moments from AIMAll, considered to be the true values. Results are shown in Figure 3c to g as *L*′ increases from 0 to 4. For brevity, only root-mean-square errors (RMSE) have been shown in the form of heatmaps. The heatmaps do not vary substantially across the different *L*′, meaning that nearly all the electrostatic error comes overwhelmingly from the charges, and contributions from higher multipole moments are largely insignificant. For *L*′ = 4, the RMSE ranges from the largest 2.88 kJ mol^–1^ for carbon–carbon to the smallest 0.04 kJ mol^–1^ for a distant hydrogen–hydrogen electrostatic interaction and taking values in this range for any other atom pairs.

Another performance test involves a FFLUX optimization of the minimum-energy monomer and a comparison to the GAUSSIAN B3LYP/aug-cc-pVTZ equivalent at the training model level of theory, shown in Table 1. For the urea model featured in this work, FFLUX recovered the monomer minimum with the single point energy value of 591,720.8 kJ mol^–1^, a 2.42 × 10^–3^% error, or 14.3 kJ mol^–1^ difference compared with GAUSSIAN. The RMSE between the two methods is 0.045 Å, which translates to the optimized geometry maximum bond length error not exceeding 0.01 Å and bond angle error 4°. Yet, the B3LYP planarity of urea was not fully recovered. The molecular dipole moment was also calculated and compared to the training level of theory in order to test the charge and dipole models generated. In GAUSSIAN, the calculated dipole moment of the B3LYP/aug-cc-pVTZ optimized gas-phase urea monomer is 4.24 D, while FFLUX predicts the value of 4.35 D for the B3LYP reference geometry. This difference shows that the model captures the charge distribution in the molecule reasonably well. The error in the predicted energy is not as small as in some other molecules,^19,32^ but a newer version of FEREBUS^41^ has been shown to produce markedly better models using the RMSE objective function for hyperparameter optimization rather than the concentrated log-likelihood function used in this work.

**Table 1:** Single-Point Energy and Molecular Dipole Moment Predictions by FFLUX for the Urea Monomer

**monomer**	**B3LYP**	**FFLUX**	**Δ**
single-point energy (kJ mol^–1^)	–591,706.5	–591,720.8	2.42 × 10^–3^%
dipole moment (Debye)	4.24	4.35	2.59%

#### 3.1.3 FFLUX Geometry Optimization of Urea Dimers

##### 3.1.3.1 Computational Details

The current version of FFLUX uses monomers as training systems, and hence requires no parametrization when simulating single molecules. However, in order to perform MD involving more than a single molecule an extra intermolecular potential is needed. As mentioned before, such a potential consists of long-range electrostatics predicted by the GPR in the form of flexible multipole moments, as well as geometry-independent van der Waals dispersion and repulsion (correlation and exchange). Although both correlation and exchange contain the inverse distance factor, correlation and exchange cannot be multipole-expanded for a monomer because they explicitly depend on the system’s orbital overlap;^42^ therefore, intermolecular dispersion and repulsion cannot be captured by monomeric modeling, and an external parametric potential is required. This fact remains a disadvantage of the current methodology but *N*-meric modeling is current work in progress where GPR models are trained on clusters of *N* molecules. At present, *N*-meric models can only be applied to systems of the same size (*i*.*e*., a dimer model can only be used in a dimer simulation) but it is possible to extend these models to larger systems. To make the *N*-meric models applicable to systems larger than they have been trained for requires significant changes to the DL_FFLUX code and therefore represents a longer-term goal.

In parallel work on formamide^19^ a set of van der Waals, 12–6 potential parameters, originally derived by Hagler *et al.*,^43,44^ was extensively studied and eventually adapted for use in FFLUX at *L*′ = 3. This formamide-adapted set (here referred to as “adapted Hagler” (ADHAG)) was used in dimeric FFLUX runs in this work and is available in Section 3 of the SI (Table S7). To test the ADHAG parameter set, two series of dimer optimizations were performed: minimum geometry (A) and random geometry (B). For run A, the program GAUSSIAN^35^ at B3LYP/aug-cc-pVTZ level of theory with the D3 dispersion correction^45^ was used to optimize the 5 urea dimers corresponding to the energy minima of Figure 1. The optimized geometries and their energies were used as benchmark references for the FFLUX calculations. These optimized geometries were also used as starting geometries in FFLUX optimizations. For run B, a set of 75 random geometries, positioned away from the 5 minima, was generated by setting two gas-phase, B3LYP/aug-cc-pVTZ-optimized monomers at a random distance in the range of 2 to 5 Å, and at a random Euler angle. The FFLUX-optimized minima were examined quantitatively with respect to their geometry *via* the RMSD of all atoms, hydrogen bonds and single-point energies (relative energy ranking). In contrast, random geometries, also positioned away from the 5 minima, were assessed qualitatively for the convergence to the final optimized geometry with their corresponding GAUSSIAN converged structures as references.

All dimer optimizations (run A and B) were performed in a 50 Å × 50 Å × 50 Å cubic box with a Hoover thermostat relaxation constant of 0.04 ps using DL_POLY’s “zero Kelvin” (0 K) optimizer. The 0 K optimizer is effectively a low temperature MD run where the atoms are permitted to move in the direction of the calculated forces. However, the atoms’ velocities are limited to the values attainable only in a 10 K simulation. The parameter setting *L*′ = 3 was used as the highest multipole interaction rank, where all multipolar interactions between monopole (*l* = 0), dipole (*l* = 1), quadrupole (*l* = 2), and octupole moments (*l* = 3) are present. The program DL_FFLUX uses smooth particle mesh Ewald (SPME) summation for the electrostatics periodic boundary conditions and requires two parameters that handle it: (i) the Ewald convergence parameter was set to 0.000001 Å^–1^, and (ii) the dimension of the SPME charge array (the range of the reciprocal space sum) was set to “20 20 20”. Both electrostatics and 12–6 potential interaction cut-offs were set to 16 Å. SPME and periodic boundary conditions were used in these calculations purely for timing reasons. DL_FFLUX has both a “cluster” and “Ewald” mode, with the Ewald mode being much faster.

All of the optimizations were run for 20,000 timesteps (each time step being 1 fs) and final geometries were considered optimized if they met two criteria: (i) the gradient of the energy between the final step (*F*) and the *F*–1000th step should not exceed 1 × 10^–4^ kJ mol^–1^ time step^–1^, and (ii) the deviations in energy from the straight line connecting step *F* and *F*-1000 should not exceed 0.1 kJ mol^–1^. The deviation was calculated as an RMSD, comparing the energy at each of the 1000 steps to the energy at the equivalent point on the straight line connecting step *F* and *F*-1000.

##### 3.1.3.2 FFLUX Minimum-Energy Dimer Optimizations

**Figure 4:**
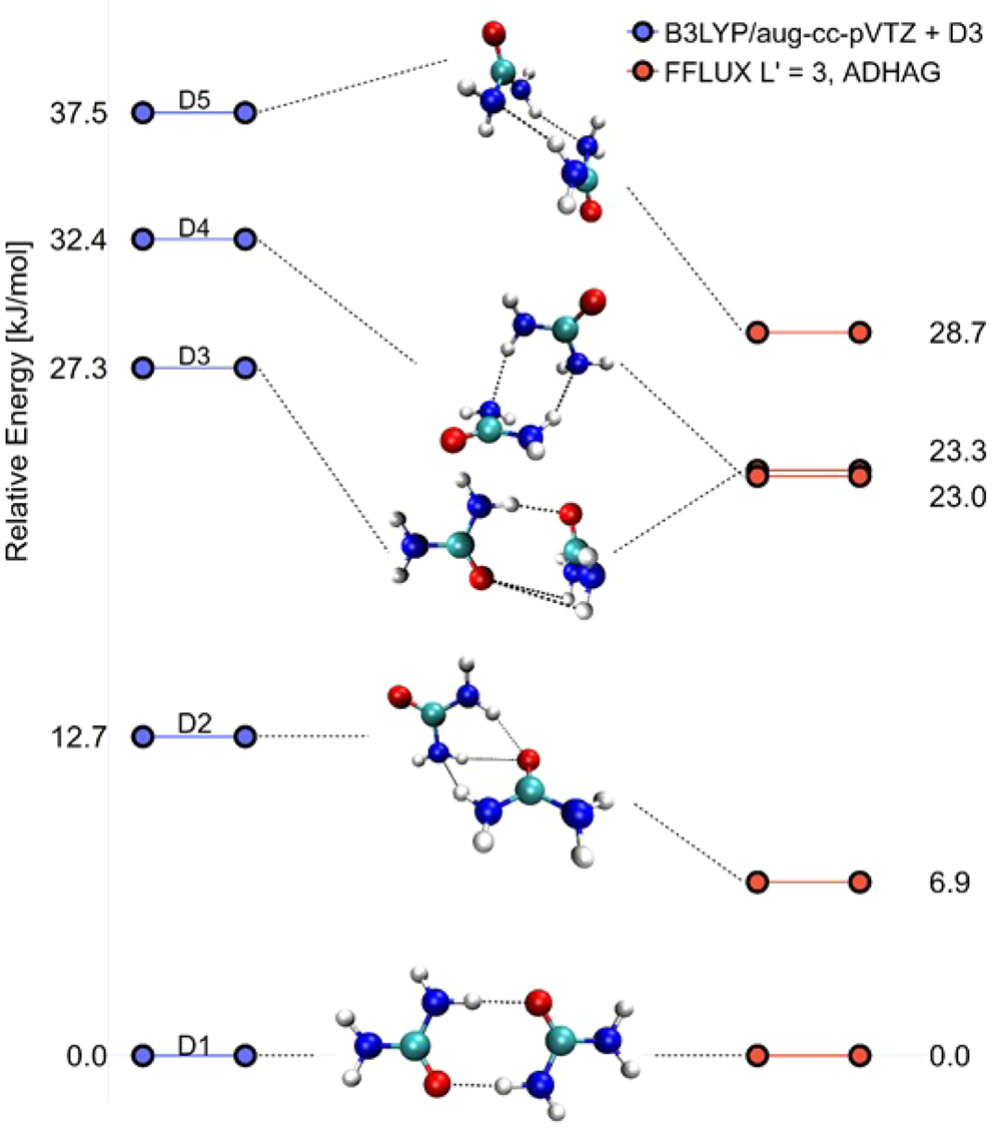
Relative energy ranking of the five urea dimers corresponding to energy minima obtained using B3LYP/aug-cc-pVTZ-D3 and FFLUX.

The final geometries of FFLUX 0 K optimized dimers, *D1*–*D5*, were benchmarked against the GAUSSIAN B3LYP-D3/aug-cc-pVTZ-optimized reference geometries and energies. Table 2 contains hydrogen bond distances and RSMD values computed using the Kabsch algorithm, while Table 3 contains hydrogen bond angles in geometries from both methods. Relative energy rankings are shown in Figure 4.

**Table 2:** Hydrogen Bond Lengths of the Five FFLUX-Optimized versus B3LYP-D3/aug-cc-pVTZ GAUSSIAN-Optimized Dimers^a^

		**hydrogen bond lengths (Å)**					
**dimer**	**method**	O···HN (1)	O···NH (2)	O···NH (3)	N···HN (1)	N···HN (2)	**RMSD (Å)**
*D1*	B3LYP-D3	1.82	1.82				
	FFLUX	1.82	1.82				0.02
*D2*	B3LYP-D3	1.97	2.53		2.13		
	FFLUX	2.01	2.66		2.07		0.08
*D3*	B3LYP-D3	2.96	2.96	1.94			
	FFLUX	3.14	2.98	1.98			0.05
*D4*	B3LYP-D3				2.24	2.24	
	FFLUX				2.17	2.17	0.04
*D5*	B3LPY-D3				2.28	2.28	
	FFLUX				2.10	2.16	0.05

a Numbers in parentheses correspond to hydrogen bond labels in Figure 1. RMSDs between FFLUX and B3LYP-D3 optimized structures are also provided.

**Table 3:** Hydrogen Bond Angles of the Five FFLUX-Optimized versus GAUSSIAN-Optimized Dimers^a^

		**hydrogen bond angles (°)**				
**dimer**	**method**	CO···H (1)	CO···H (2)	CO···H (3)	N···HN (1)	N···HN (2)
*D1*	B3LYP-D3	121.5	121.5			
	FFLUX	120.0	120.0			
*D2*	B3LYP-D3	116.6	106.2		105.6	
	FFLUX	109.5	106.4		102.4	
*D3*	B3LYP-D3	134.8	134.8	100.9		
	FFLUX	129.4	128.7	93.8		
*D4*	B3LYP-D3				94.3	94.3
	FFLUX				96.4	96.4
*D5*	B3LPY-D3				119.2	119.2
	FFLUX				120.6	123.1

a Numbers in parentheses correspond to hydrogen bond labels in Figure 1.

FFLUX accurately recovered all five dimers with no RMSD value exceeding 0.08 Å, and hydrogen bond length differences below 0.2 Å overall, and 0.1 Å in 70% of cases (Table 2). The average and maximum errors in the hydrogen bond angles (Table 3) are at 3.5 and 7.1° (for D2), respectively. It should be noted that such an accurate recovery of hydrogen bond features is primarily due to the presence of higher multipole moments in the intermolecular potential (as shown in Figures S33–S37 in Section 4 of the SI). Optimization runs at *L*′ = 0 and 1 did not sustain the geometries present in the *ab initio* GAUSSIAN-optimized dimers. The dimers disintegrated to form chemically irrelevant structures lacking the expected hydrogen bonding. It was only at *L*′ ≥ 2 that the *ab initio* hydrogen bonds were recovered qualitatively (*i*.*e*., dimers visually indistinguishable from *ab initio*), and quantitatively at *L*′ = 3, as indicated in Tables 2 and 3. The use of *L*′ = 4 did not further improve the RMSD of the geometries optimized at *L*′ = 3. This is unsurprising because the nonbonded potential was, as mentioned before, adapted for use at the particular value of *L*′ = 3. The recovery could be somewhat improved by tweaking the 12–6 potential parameters specifically for this system but there is a point of diminishing returns to this exercise. The nonbonded potential itself is merely an approximation to the true dispersion and repulsion in the first place.

FFLUX also managed to recover well the relative energy ranking of the five dimers (with the global minimum dimer *D1* used as the reference point) as shown in Figure 4. FFLUX agrees with the B3LYP-D3/aug-cc-pVTZ counterpart aside from a crossover (barely visible in the figure) of dimers *D3* and *D4*. FFLUX underestimated the energy difference between these two dimers, predicting it at only 0.3 kJ mol^–1^ compared to the 5.1 kJ mol^–1^ seen in the GAUSSIAN calculations. The energies were both underpredicted, but to a different extent thereby causing the crossover. The largest relative energy discrepancy was for dimer *D4*, just less than 10 kJ mol^–1^.

A common phenomenon in amides upon their formation of hydrogen bonds, for example, when transitioning from the gas to solid phase,^46^ is a change in C=O and C–N bond lengths. Table 4 shows changes in the C=O and C–N urea bond lengths upon hydrogen bond formation. The symbol Δ denotes the difference between bond lengths in either the B3LYP-D3/aug-cc-pVTZ optimized monomer and B3LYP-D3/aug-cc-pVTZ dimers, or FFLUX-optimized monomer and FFLUX-optimized dimers. The bond length changes, albeit extremely subtle, agree to the sign, but more importantly, to the magnitude of the change.

**Table 4:** C=O and C–N Bond Length Changes upon Dimer Formation of Hydrogen Bonds

	C=O **distance/Å**				**C–N distance/Å**			
**dimer**	B3LYP	Δ	FFLUX	Δ	B3LYP	Δ	FFLUX	Δ
***D1***	1.233	0.014	1.229	0.010	1.381	0.009	1.379	0.007
***D2***	1.231	0.012	1.228	0.009	1.374	0.002	1.371	0.001
***D3***	1.225	0.006	1.222	0.003	1.384	0.012	1.379	0.007
***D4***	1.215	–0.004	1.215	–0.004	1.405	0.033	1.396	0.024
***D5***	1.215	–0.004	1.215	–0.004	1.401	0.029	1.398	0.026

#### 3.1.4 FFLUX Random Dimer Optimizations

To further explore the predictive potential of FFLUX, 75 random dimer geometry optimizations were carried out, and juxtaposed with equivalent B3LYP-D3/aug-cc-pVTZ optimizations. For the FFLUX runs the same procedure as described in Section 3.1.3.1 was applied, while DFT optimizations were performed by using GAUSSIAN, initially using steepest descent before being refined with the default Newton–Raphson algorithm.

**Figure 5:**
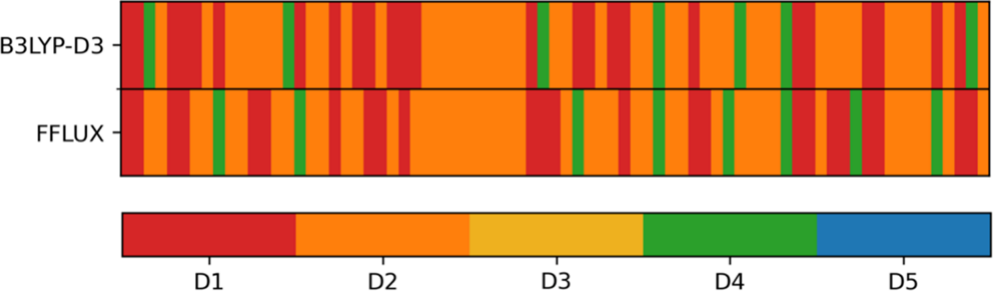
Convergence of 75 random dimers (each one marked by a thin vertical stripe) obtained with FFLUX versus GAUSSIAN with a 68% rate of success. The degree of convergence is measured by counting the number of stripes that have a matching color between B3LYP-D3 and FFLUX.

The final FFLUX structures were classified as converged if their RMSD’s were below 0.125 Å off the geometrically closest *ab initio* optimized minimum dimer. This threshold was selected based on the own maximum variance within the GAUSSIAN-optimized reference set of geometries itself and all dimer types considered. Moreover, if a random dimer converged to a geometric isomer of a known minimum dimer (*e*.*g*., enantiomers of dimer *D2*, where the NH_2_ groups are inverted), it was considered converged too. As shown in Figure 5, 51 of 75 random dimers converged to the qualitatively same dimer for both FFLUX and GAUSSIAN with the average RMSD of 0.09 Å for the former, well within the accepted threshold.

### 3.2 Effect of Using Flexible *versus* Static Multipole Moments

**Figure 6:**
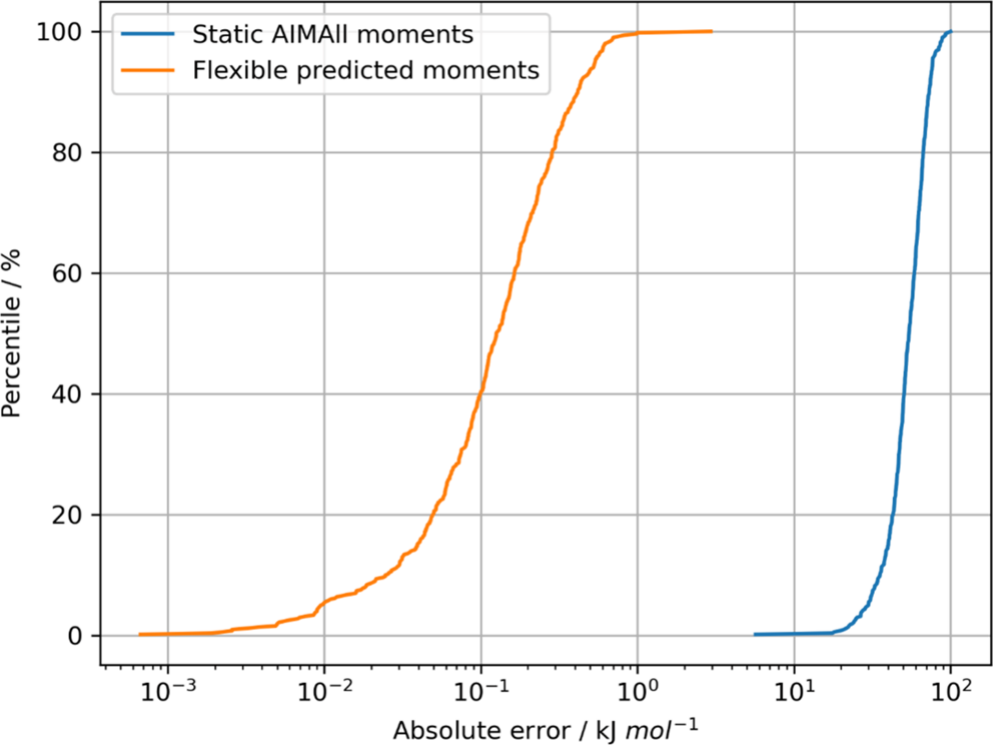
S-curves showing the error in total intermolecular electrostatic energy when using predicted flexible multipole moments (orange) and static multipole moments (blue) at *L*′ = 4.

An important feature of FFLUX is the flexibility of its multipole moments, *i*.*e*., they change depending on molecular geometry allowing intramolecular polarization and charge transfer effects to be captured.^47^ To showcase the benefits of this attribute, the 500 dimeric geometries of the electrostatics test in Section 3.1.1 were equipped with static moments based on the B3LYP/aug-cc-pVTZ optimized urea monomer geometry and its AIMAll-calculated multipole moments. We note in passing that the exact tensor component values are available in Section 6 of the SI. Then, the total system’s intermolecular electrostatic energy was calculated for each geometry at *L*′ = 4 using DL_FFLUX while errors were evaluated against the energies associated with the true AIMAll moments used in the run described in Section 3.1.1Figure 6 shows the S-curves of these errors, as well as the FFLUX electrostatic energy prediction errors, also from Section 3.1.1 but for the total system energy rather than individual atom’s energy. The *flexible* FFLUX-predicted energies (orange) perform significantly better than the energies from the *static* moments (blue), with a mean error of just over 0.1 kJ mol^–1^ and a maximum error of 3 kJ mol^–1^. Calculations using the static moments have errors over 2 orders of magnitude larger, with a mean error of about 50 kJ mol^–1^ and a maximum error of 100 kJ mol^–1^. The magnitudes of these errors clearly indicate the importance of flexible multipole moments if one strives to achieve better accuracy in MD.

## 4 Conclusions

This work featured for the first time a novel Quantum Chemical Topology, Gaussian process regression (GRP) force field FFLUX in the application to urea monomer and dimers. The GPR model, trained with 2000 geometries at the B3LYP/aug-cc-pVTZ level of theory, achieved a mean absolute error of 0.4 kJ mol^–1^ and a maximum error below 7.0 kJ mol^–1^. The optimization of the monomer gave an error of 2.4 × 10^–3^% in the energy and 2.6% in the dipole moment showing the ability of FFLUX to predict intramolecular energies with reasonable accuracy. The test of multipole moments turned up a maximum error of 2.9 kJ mol^–1^ for the carbon–carbon electrostatic interaction, and the minimum error of 0.04 kJ mol^–1^ for a hydrogen–hydrogen electrostatic interaction at *L*′ = 4, the interaction rank involving all possible multipole interactions between a pair of atoms up to and including hexadecapole. This is quite a feat as charges of carbon atoms surrounded by three electronegative atoms are extremely labile to conformational changes. Another benchmark, the FFLUX geometry optimizations of the five minimum-energy urea dimers were performed using the urea model and a nonbonded potential fine-tuned in a previous work on formamide. The dimer geometries were recovered with a root-mean-square deviation below 1.0 Å, and the correct C=O and C–N bond length behavior on hydrogen bond formation. The energy ranking of the recovered geometries agreed with the B3LYP-D3 equivalent with an exception of a small crossover between two consecutive dimers. A set of 75 random dimers away from equilibrium geometries was used to further examine the predictive potential of FFLUX. It turned out that 68% of them converged to the qualitatively same minima as the *ab initio* counterparts.

Ongoing research explores the possibility of dimeric and multimeric GPR training with the main goal to ultimately rid FFLUX of the burden of nonbonded parameters. However, the greatest challenge in this case proved to be the robustness of the urea models. Unlike formamide, which was very successfully modeled in our previous work,^19,20^ urea is a much more flexible molecule possessing a larger conformational space such that the well-sampledness of it becomes essential. The classical MD used for geometry generation in this work has shown to approach its capability limits; the urea models could not be successfully trained on a par with the formamide ones. We hypothesize that the patch of the potential energy surface sampled by a classical force field overlaps ever poorer with the *ab initio* potential energy surface with increasing system dimension and molecular flexibility, forcing the GPR to extrapolate. Some investments have been also made in the direction of a general improvement of the training itself, *i*.*e*., the implementation of multiple metaheuristic optimizers, as well as a redefinition of the objective function.^41^ If progress is made in these areas, accurate simulations of larger clusters will become feasible for a larger number of systems, with the crystal simulations being one of the ultimate goals of FFLUX.

## Data Availability

The data that support the findings of this study are available from the corresponding author upon request.

## Supporting Information Available

Coordinates of the B3LYP/aug-cc-pVTZ optimized monomer (Table S1) and dimers (Tables S2–S6) with corresponding images (Figures S1–S6); Bundled S-curves (Figure S7); S-curves for the raw predictions of multipole moment tensor components (Figures S8–S32); ADHAG nonbonded potential parameter set (Table S7); RMSD plots of FFLUX-optimized dimers against the B3LYP equivalent (Figures S33–S37); Urea training set mist plot (Figure S38); Multipole moment tensor components for the FFLUX static moments run (Tables S8–S15). (PDF)
