# Supplementary material for: Toward Gaussian Process Regression Modeling of a Urea Force Field
Source: J Phys Chem A. 2024 Sep 20;128(39):8551–60. doi: 10.1021/acs.jpca.4c04117 (PMC11457224; doi:10.1021/acs.jpca.4c04117)
Supplement: Supplementary file 1 — jp4c04117_si_001.pdf [file jp4c04117_si_001.pdf]

Supporting Information

Towards Gaussian Process Regression modelling of a Urea Force Field

Tomasz Bukowy[a], Matthew L. Brown[a], and Paul L. A. Popelier[a]*

[a]Department of Chemistry, University of Manchester, Manchester M13 9PL, Great Britain

*E-mail: pla@manchester.ac.uk

Contents

1. Coordinates of the B3LYP/aug-cc-pVTZ optimised monomer and dimers
2. Additional S-curves:
    a. Bundled S-curves
    b. Predictions of individual AIMAll multipole moment tensor components
3. ADHAG non-bonded parameters
4. FFLUX dimer optimisation RMSD against the GAUSSIAN B3LYP/aug-cc-pVTZ optimised equivalent
5. Training set mist plot
6. Multipole moment tensor components for the FFLUX static moments run
7. References

1. Coordinates of the B3LYP/aug-cc-pVTZ optimised[1] monomer and dimers.*

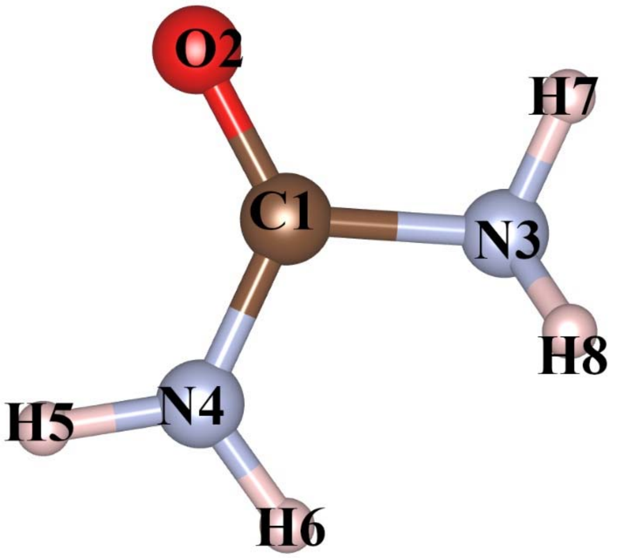

Figure S1. Structure of the urea monomer.

Table S1. Coordinates of the urea monomer optimised at the B3LYP/aug-cc-pVTZ level of theory.

Atom	X / Å	Y / Å	Z / Å
C1	-0.6721990	-2.3616090	-0.0381360
O2	-0.5199570	-2.1314660	-1.2254880
N3	-0.0130960	-3.3797720	0.6033760
N4	-1.5146230	-1.6206770	0.7517470
H5	-2.012696	-0.8706770	0.310808
H6	-1.6594400	-1.7926820	1.7283840
H7	0.6099760	-3.9394080	0.0524040
H8	-0.1219260	-3.5886590	1.5774900

* The order of atoms in the dimers follows the monomeric pattern.

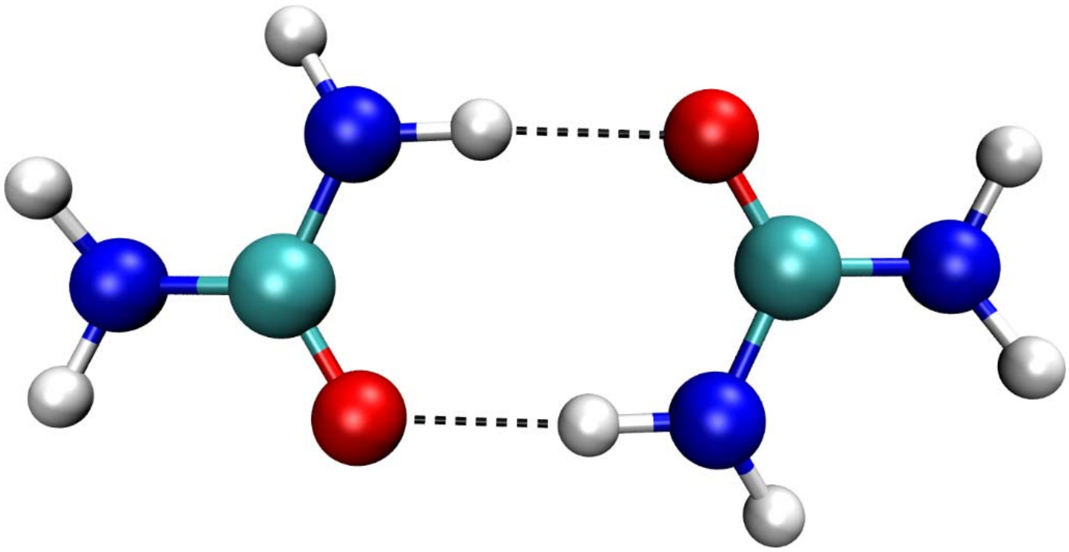

Figure S2. Structure of urea dimer D1.

Table S2. Coordinates of urea dimer D1 optimised at the B3LYP/aug-cc-pVTZ level of theory.

Atom	X / Å	Y / Å	Z / Å
C1	2.026099	0.079205	-0.002401
O2	1.403029	1.142146	0.045247
N3	3.396464	0.077520	-0.171734
N4	1.434483	-1.134236	0.089501
H5	0.411321	-1.175567	0.082633
H6	1.942781	-1.963147	-0.159280
H7	3.828203	0.974576	-0.030433
H8	3.926886	-0.713915	0.149982
C9	-2.026070	-0.079218	-0.002390
O10	-1.403001	-1.142161	0.045209
N11	-3.396436	-0.077522	-0.171695
N12	-1.434444	1.134217	0.089531
H13	-0.411278	1.175541	0.082636
H14	-1.942741	1.963142	-0.159217
H15	-3.828176	-0.974574	-0.030366
H16	-3.926845	0.713919	0.150028

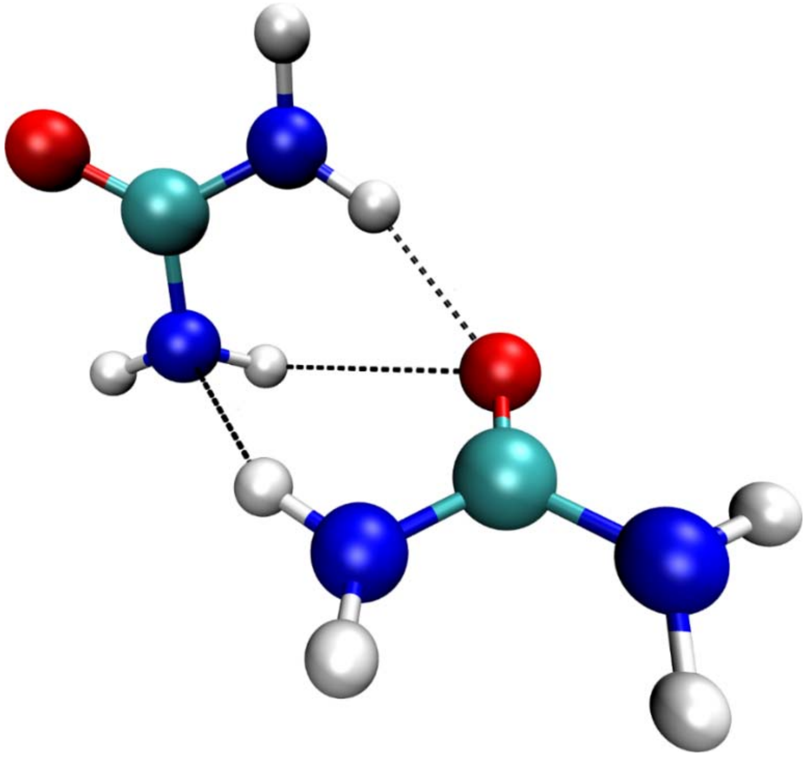

Figure S3. Structure of urea dimer D2.

Table S3. Coordinates of urea dimer D2 optimised at the B3LYP/aug-cc-pVTZ level of theory.

**Atom**	**X / Å**	**Y / Å**	**Z / Å**
C1	-1.894638	-0.022059	0.051435
O2	-1.273760	0.803691	0.720865
N3	-3.192793	0.203490	-0.337929
N4	-1.346501	-1.199736	-0.356253
H5	-0.356323	-1.321227	-0.175724
H6	-1.762070	-1.728927	-1.101282
H7	-3.638924	0.982708	0.114121
H8	-3.786950	-0.571964	-0.574660
C9	2.110868	0.090233	-0.061869
O10	3.169526	-0.206475	-0.587015
N11	1.449121	-0.784736	0.810438
N12	1.446066	1.259048	-0.275129
H13	1.804165	1.849596	-1.003067
H14	0.484861	1.360576	0.027751
H15	2.072365	-1.478110	1.192761
H16	0.836682	-0.354237	1.489444

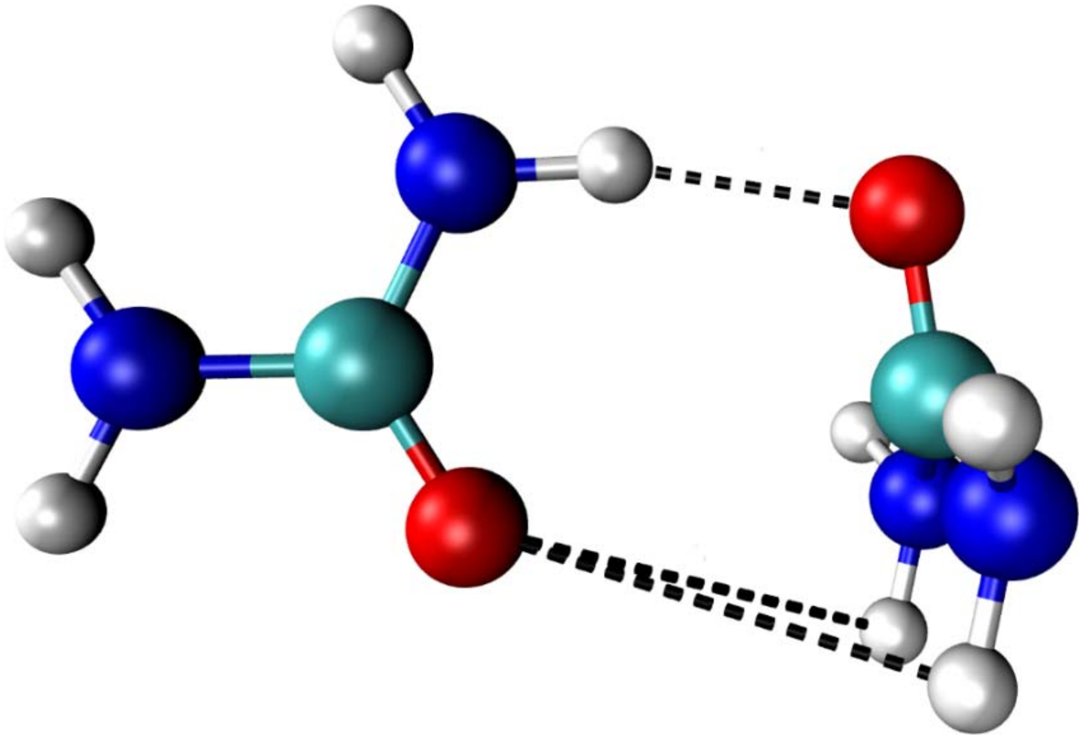

Figure S4. Structure of urea dimer D3.

Table S4. Coordinates of urea dimer D3 optimised at the B3LYP/aug-cc-pVTZ level of theory.

Atom	X / Å	Y / Å	Z /Å
C1	0.170896	1.759858	0.002703
O2	1.078804	0.937336	-0.010988
N3	0.419294	3.112830	-0.153778
N4	-1.142597	1.425949	0.162522
H5	-1.378438	0.439337	0.095749
H6	-1.856025	2.082097	-0.100893
H7	1.388358	3.362861	-0.054606
H8	-0.235475	3.774444	0.227834
C9	-0.220734	-1.777475	-0.030425
O10	-1.412901	-1.480377	-0.064069
N11	0.454076	-1.952017	1.152088
N12	0.494151	-2.043455	-1.169300
H13	0.050637	-1.712063	-2.008951
H14	1.492243	-1.919378	-1.145454
H15	-0.033761	-1.565936	1.943035
H16	1.443689	-1.767331	1.154533

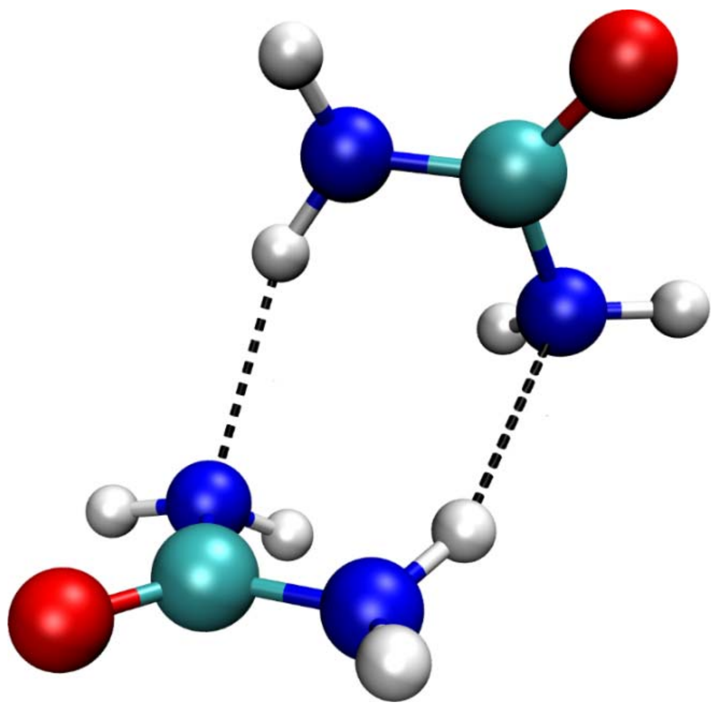

Figure S5. Structure of urea dimer D4.

Table S5. Coordinates of urea dimer D4 optimised at the B3LYP/aug-cc-pVTZ level of theory.

Atom	X / Å	Y / Å	Z / Å
C1	1.917452	-0.099388	0.187089
O2	2.740537	-0.906424	-0.196229
N3	1.618513	1.058583	-0.550813
N4	1.186864	-0.223858	1.337063
H5	1.253868	-1.124283	1.780410
H6	0.289343	0.239586	1.393637
H7	2.333835	1.248354	-1.236573
H8	1.379937	1.871515	-0.002250
C9	-1.917503	-0.099745	-0.186771
O10	-2.740556	-0.905224	0.199873
N11	-1.618331	1.061069	0.546563
N12	-1.186869	-0.228987	-1.336194
H13	-1.253992	-1.131192	-1.775888
H14	-0.289268	0.234070	-1.394622
H15	-2.333609	1.253659	1.231582
H16	-1.379638	1.871789	-0.005210

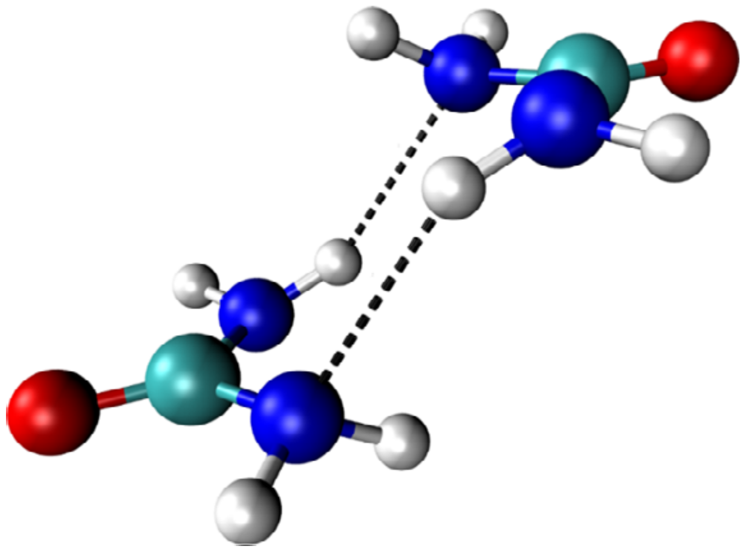

Figure S6. Structure of urea dimer D5.

Table S6. Coordinates of urea dimer D5 optimised at the B3LYP/aug-cc-pVTZ level of theory.

Atom	X / Å	Y / Å	Z / Å
C1	2.253118	-0.002341	0.160360
O2	3.311319	-0.258137	-0.378774
N3	1.391837	-1.004710	0.626602
N4	1.780177	1.268408	0.362721
H5	2.315975	1.991114	-0.086643
H6	0.781769	1.416136	0.420752
H7	1.864830	-1.894076	0.683547
H8	0.879501	-0.779963	1.467201
C9	-2.146519	0.199213	-0.052508
O10	-3.205507	0.455047	0.485071
N11	-1.284749	-1.201533	-0.517927
N12	-1.673067	-1.071533	-0.253695
H13	-2.209341	-1.794146	0.195252
H14	-0.674541	-1.219065	-0.310334
H15	-1.757786	2.090807	-0.575880
H16	-0.771154	0.976538	-1.357689

2. Additional S-curves

2.1 Bundled S-curves

Figure S2.1 shows 100, 20, 10, and 2 random subsamples of the 10,000-point validations set of the following corresponding sizes: 100, 500, 1000 and 5000. This computational experiment shows that reporting a maximum error (100% percentile) is not a perfect metric because it depends on the size and diversity of the validation set, which is indicated by the increasing spread of the S-curves with the decreasing validation set size. For example, the spread of the 100-point validation set S-curves (orange) is much larger than that of tight bundle of the 5000-point validation set S-curves (magenta), especially at the 100% percentile. This effect has to do with the increased presence of outliers that evermore push the 100% percentile to the right.

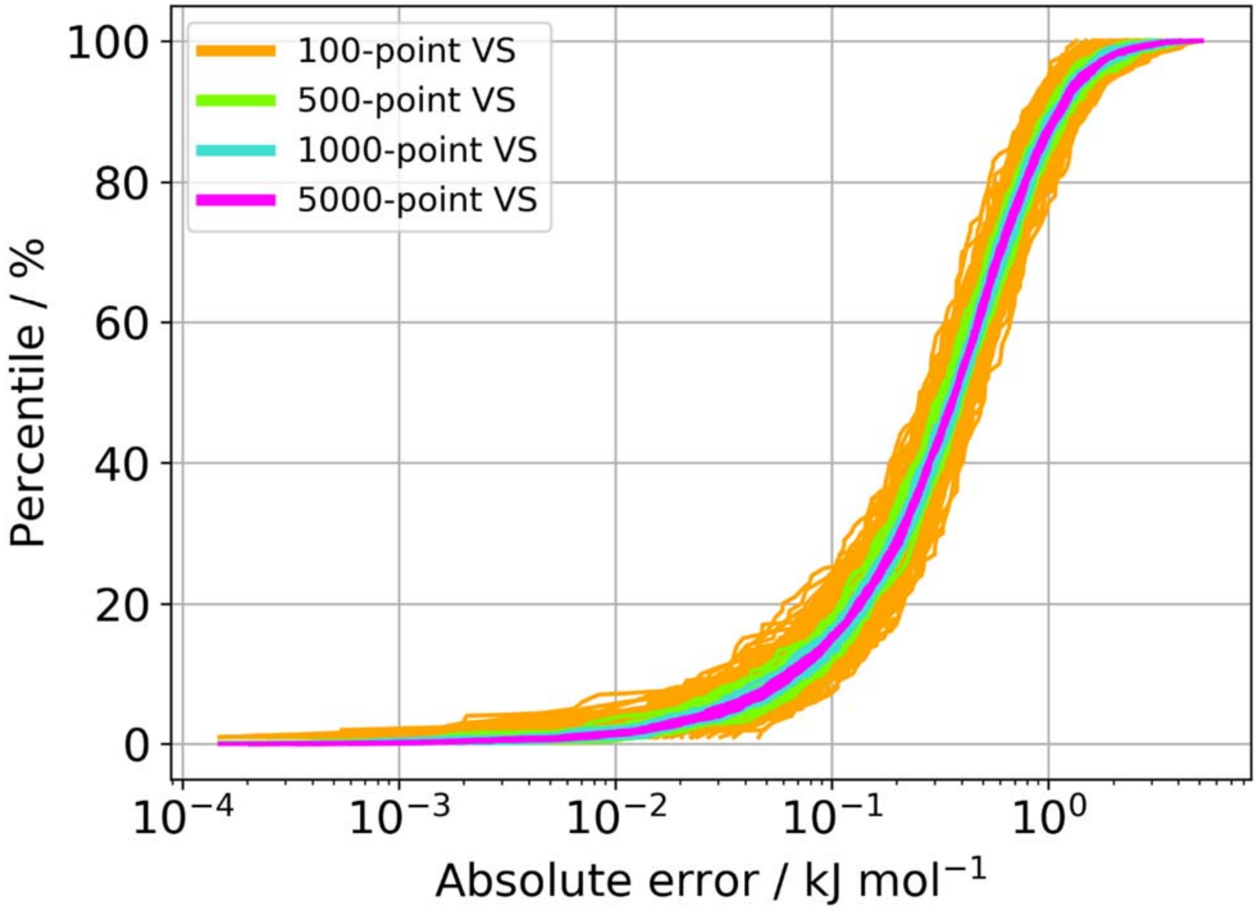

Figure S7. Bundled S-curves showing the maximum error spread across validation sets.

2.2 S-curves for the predictions of individual AIMAll multipole moment tensor components.

The components of multipole tensors are denoted Qlm, Qlmc or Qlm$s$, where the indices are sometimes written as subscript but not here because of convenience in the labelling of all plots in this section. The index l is the rank of the multipole with $m = 2l + 1$ independent tensor components, while indices c and s loosely refer to "cosine" and "sine", which represent two routes to make the original, complex exp(imφ)-like expression real. For example, in the case of the dipole moments, the indices c and s respectively lead to the x and y components. The z component is always related to the m-0 index. The S-curves were generated using a 500-point validation set of dimeric geometries sampled from a 300 K CP2K simulation. The units of multipole moments featured in the figures below are $e \times Bohr^l$, where l is the exponent of the Bohr unit. For example, for charge we have $l = 0$, and the units simply to $e \times Bohr^0 = e$.

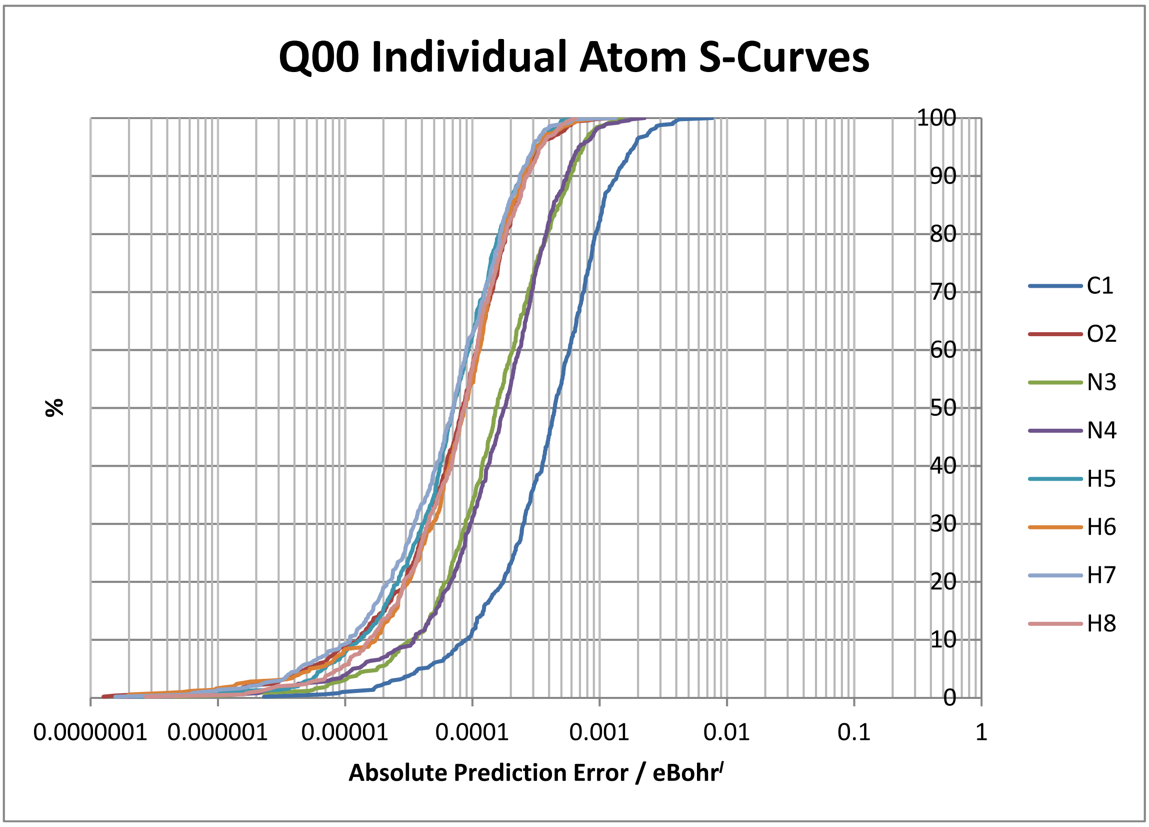

Figure S8. Q00 individual atom S-curves.

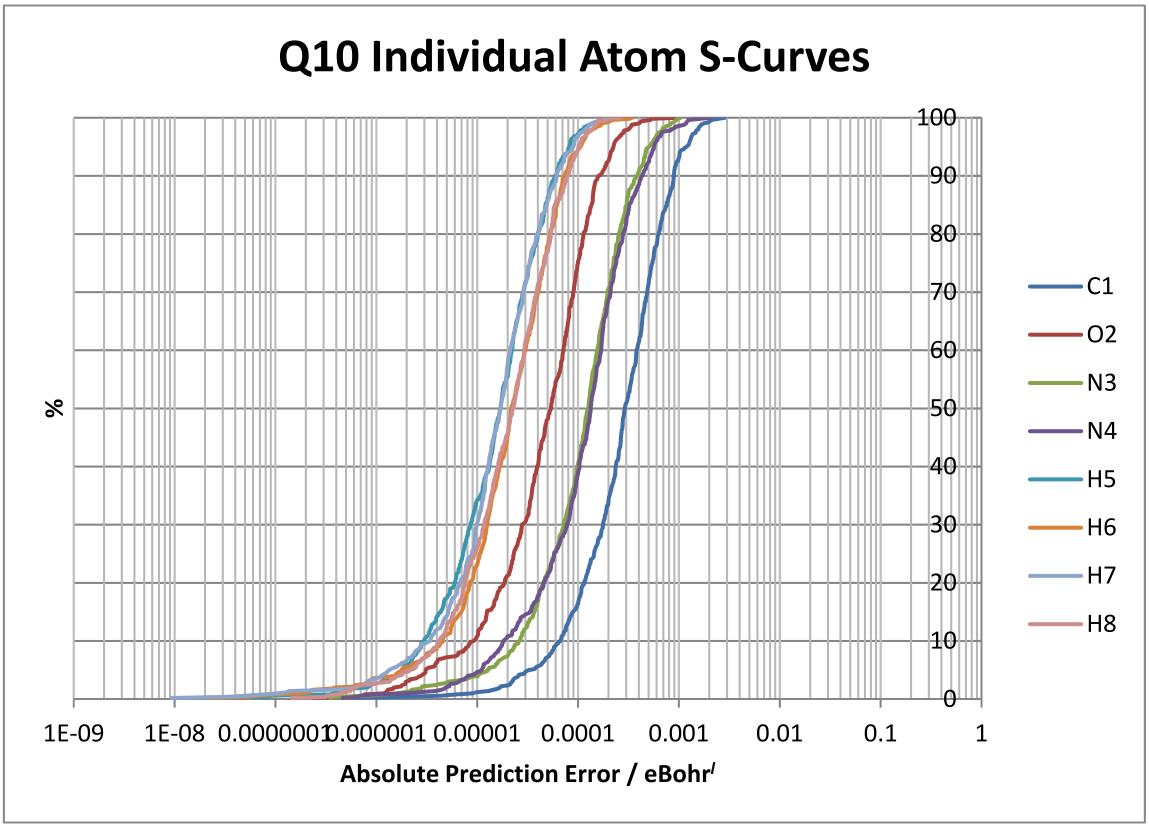

Figure S9. Q10 individual atom S-curves.

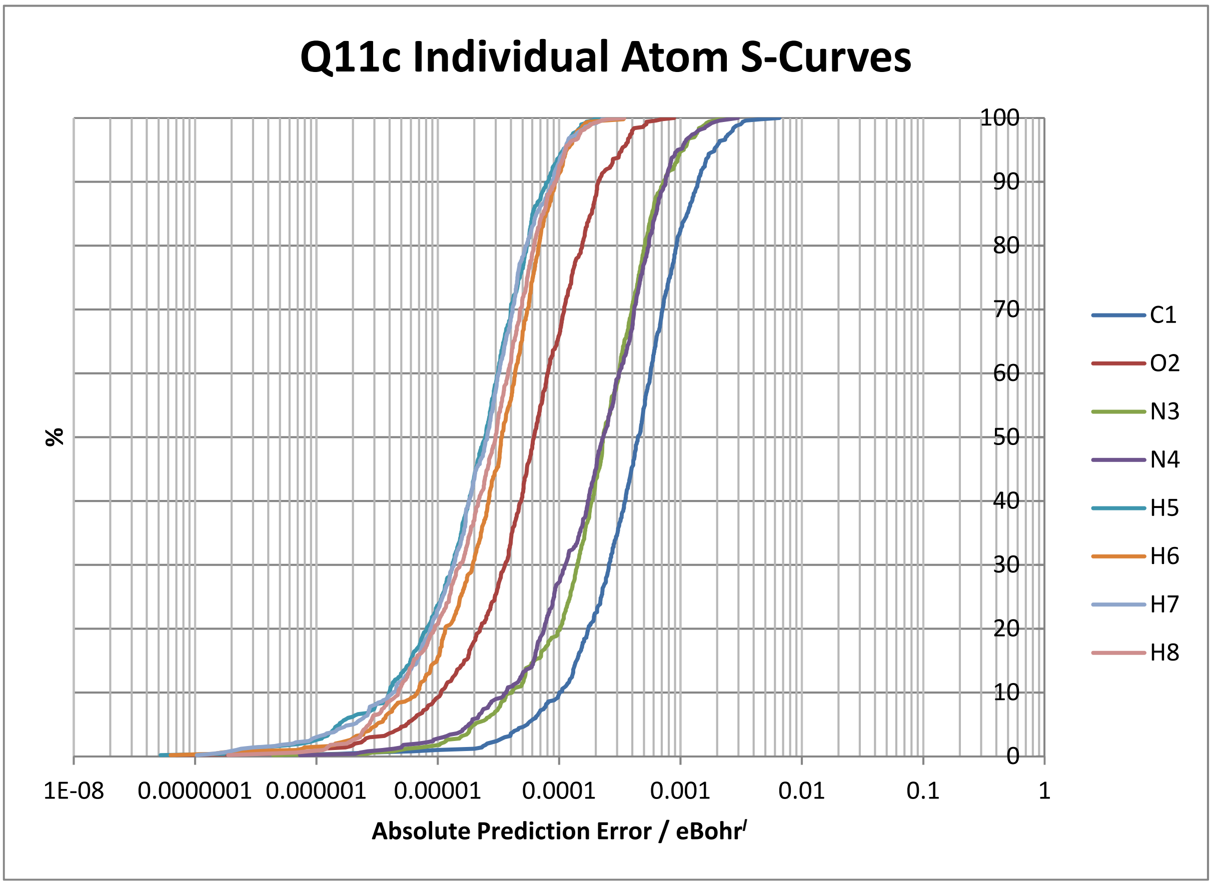

Figure S10. Q11c individual atom S-curves.

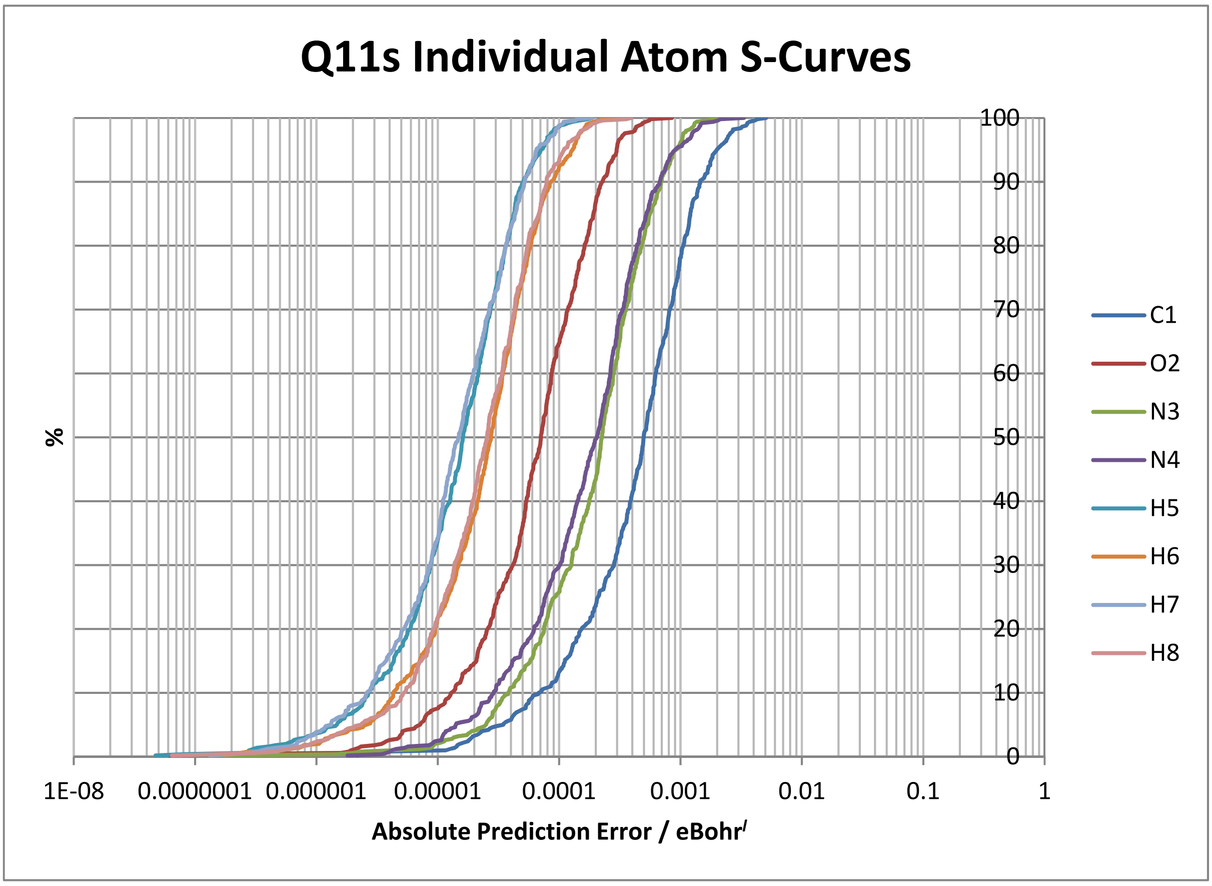

Figure S11. Q11s individual atom S-curves.

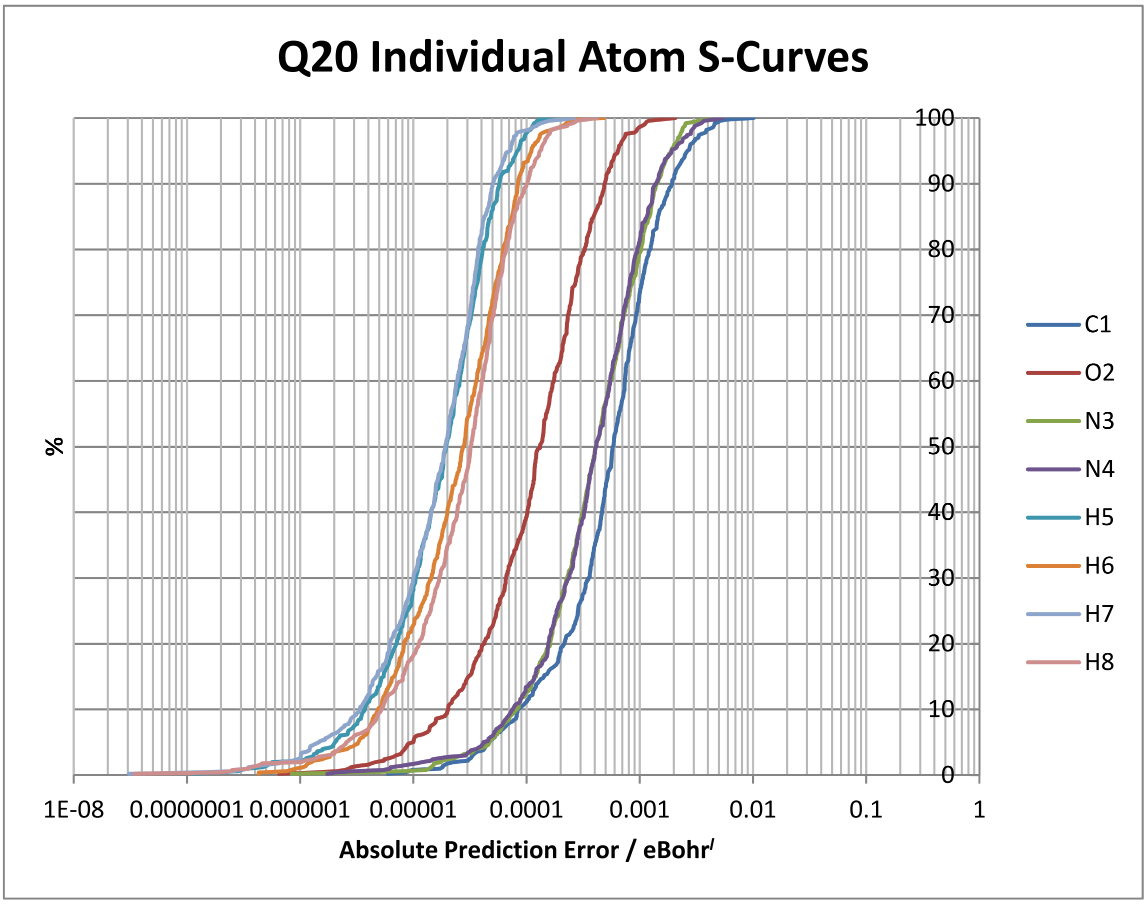

Figure S12. Q20 and individual atom S-curves.

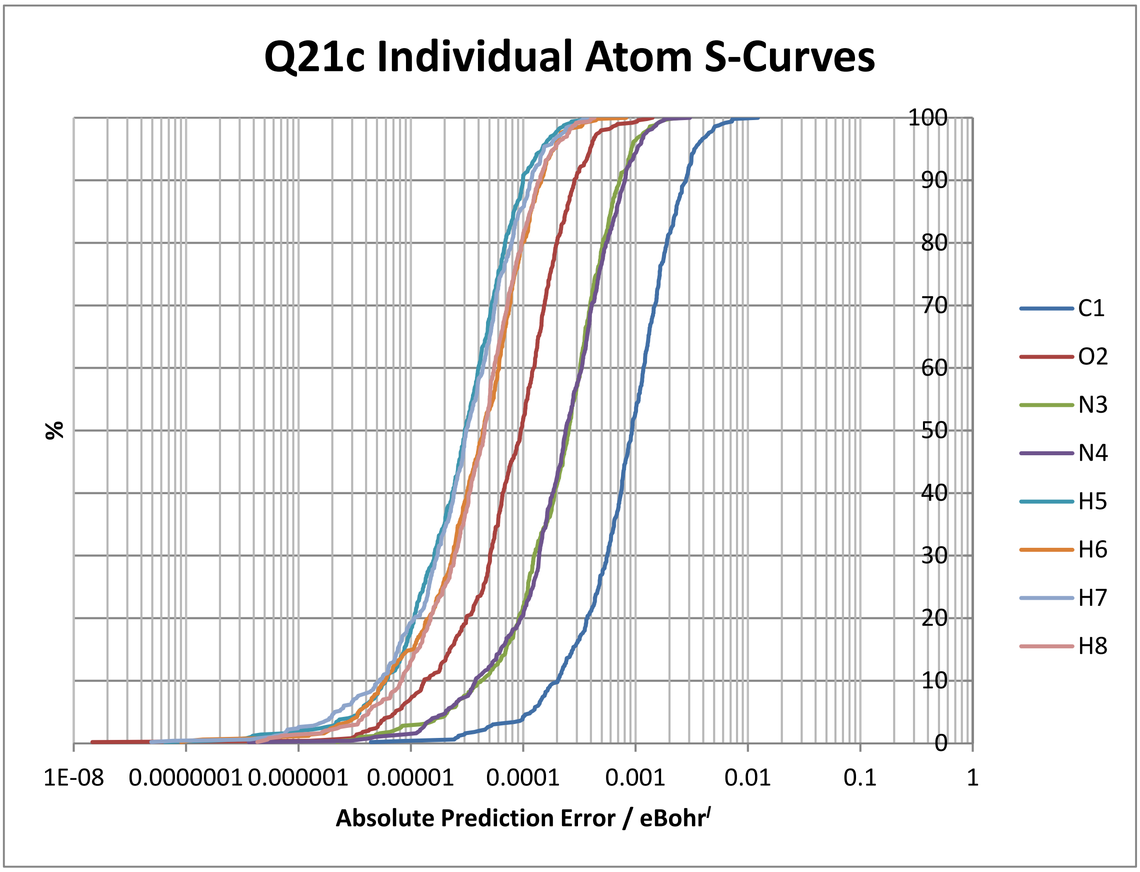

Figure S13. Q21c individual atom S-curves.

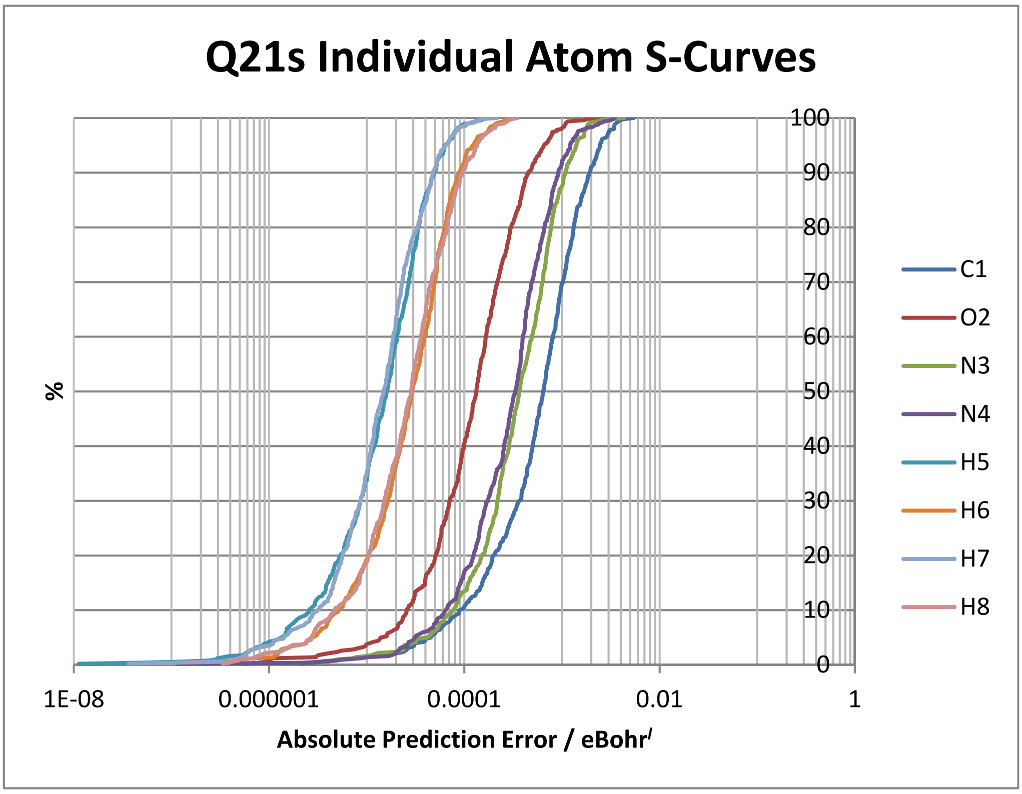

Figure S14. Q21s individual atom S-curves.

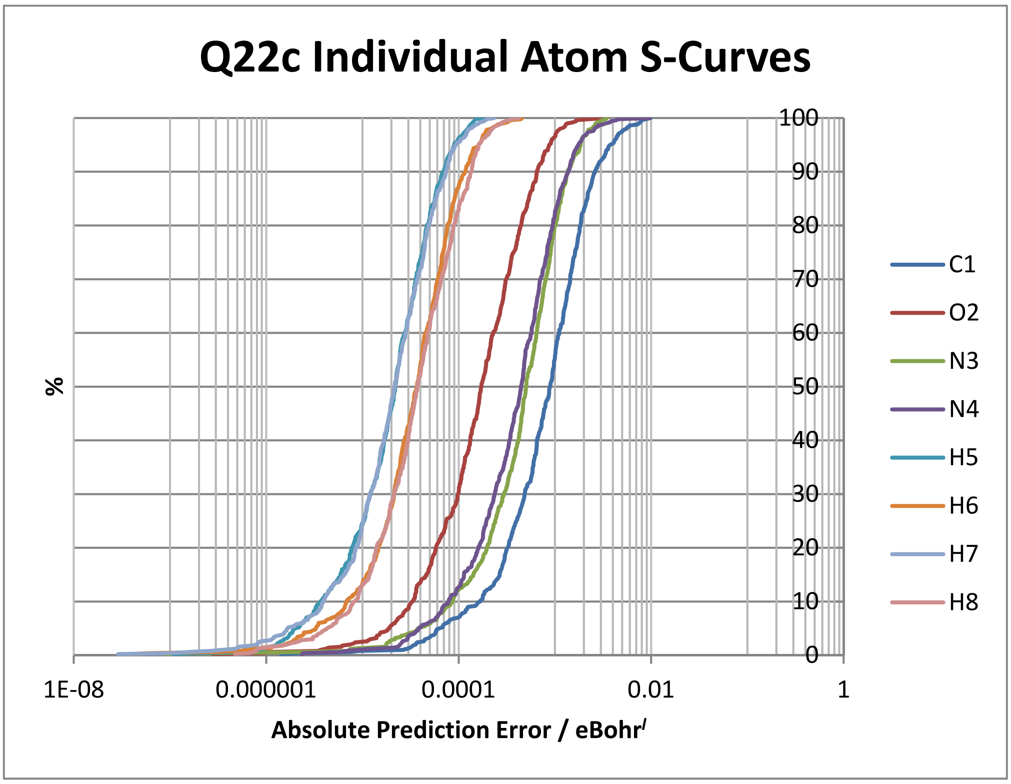

Figure S15. Q22c individual atom S-curves.

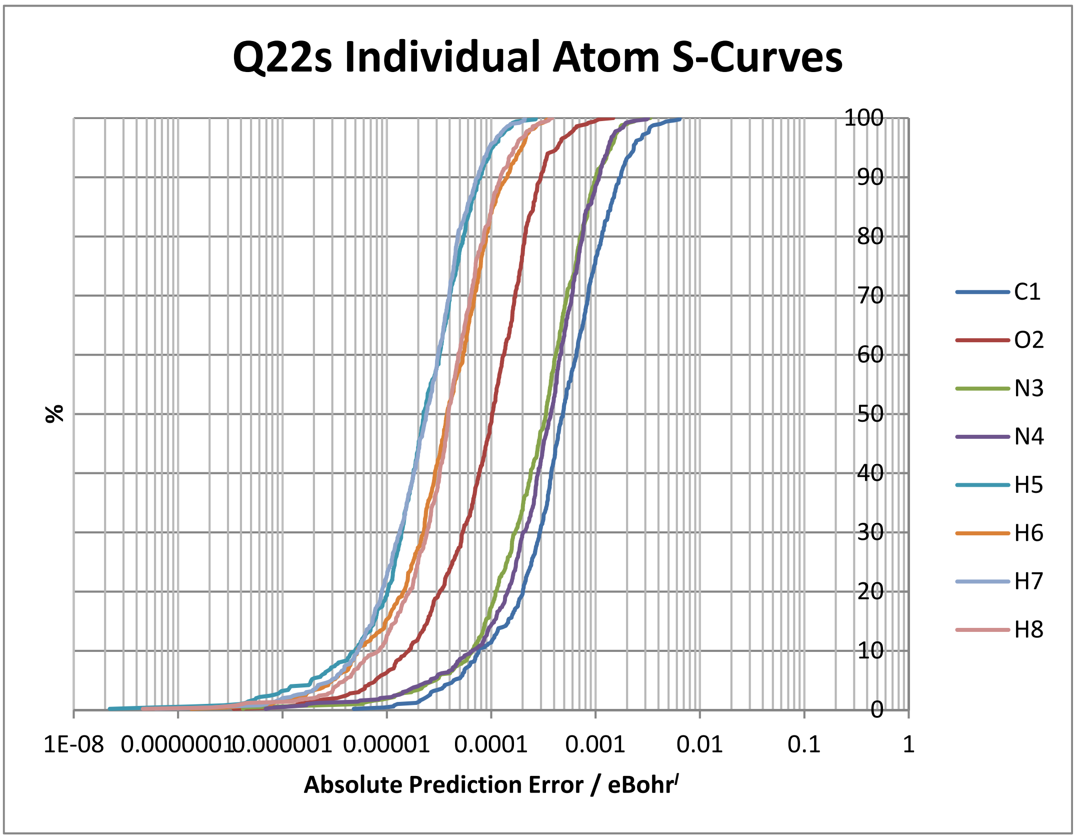

Figure S16. Q22s individual atom S-curves.

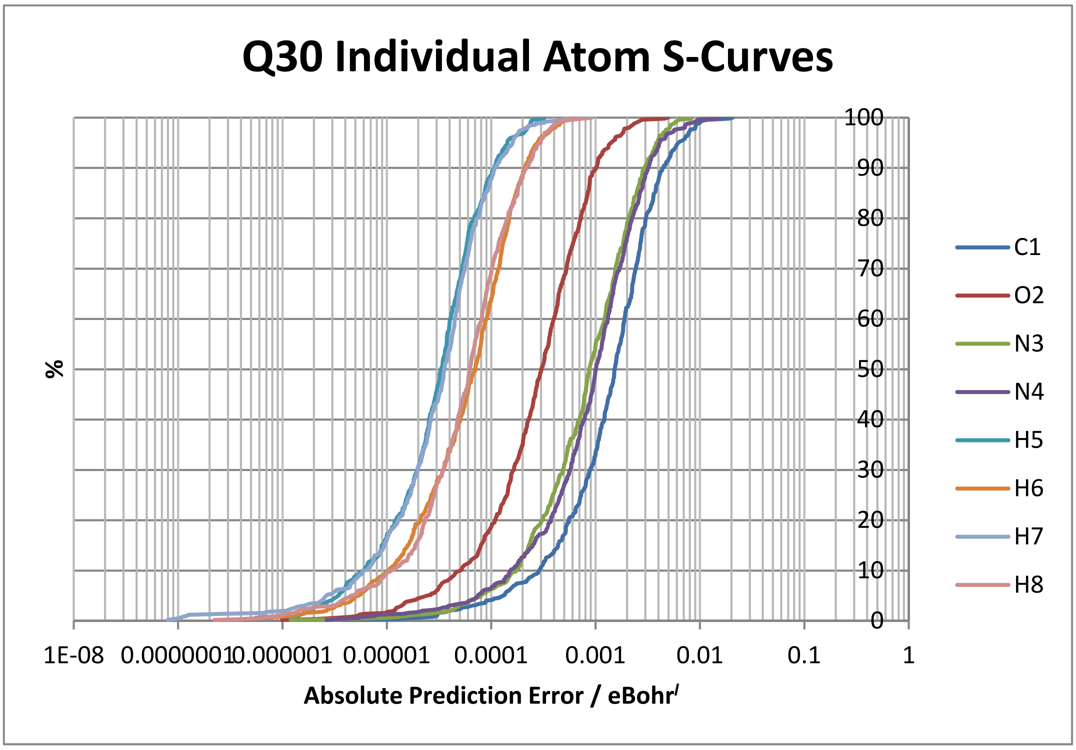

Figure S17. Q30 individual atom S-curves.

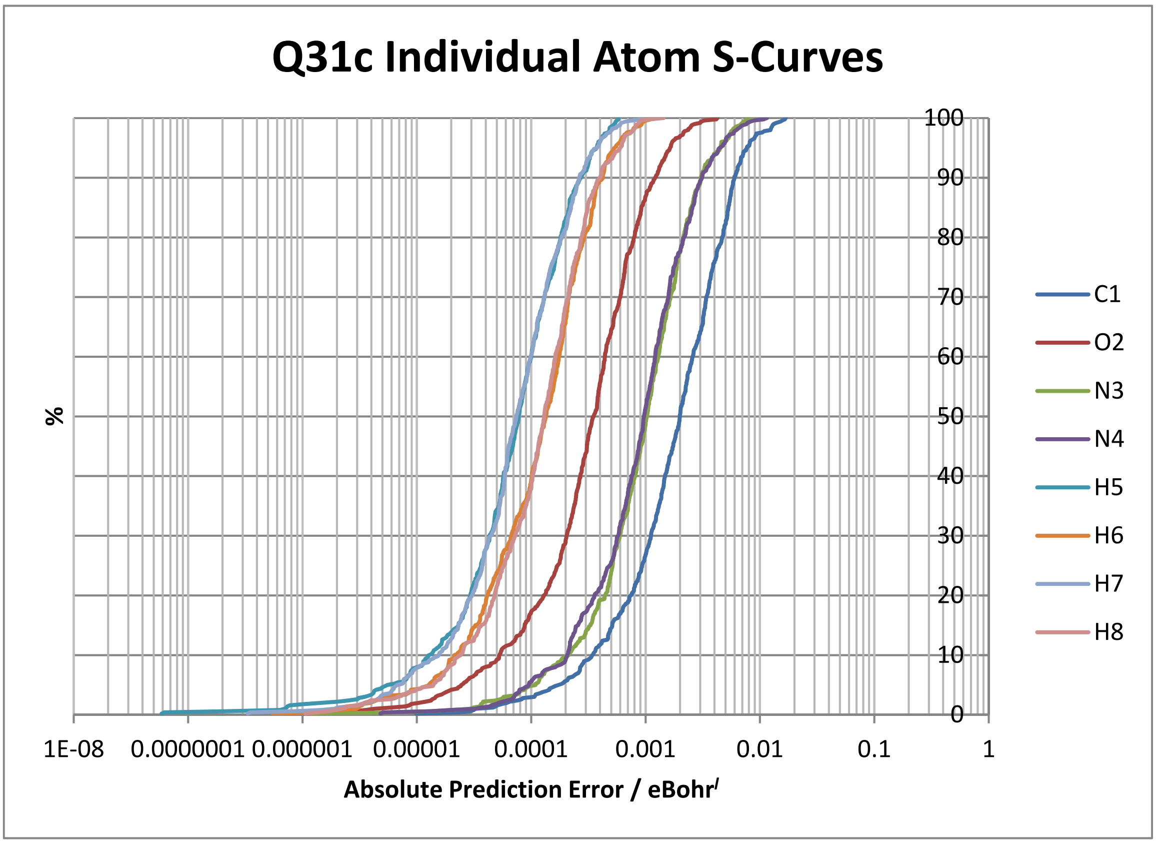

Figure S18. Q31c individual atom S-curves.

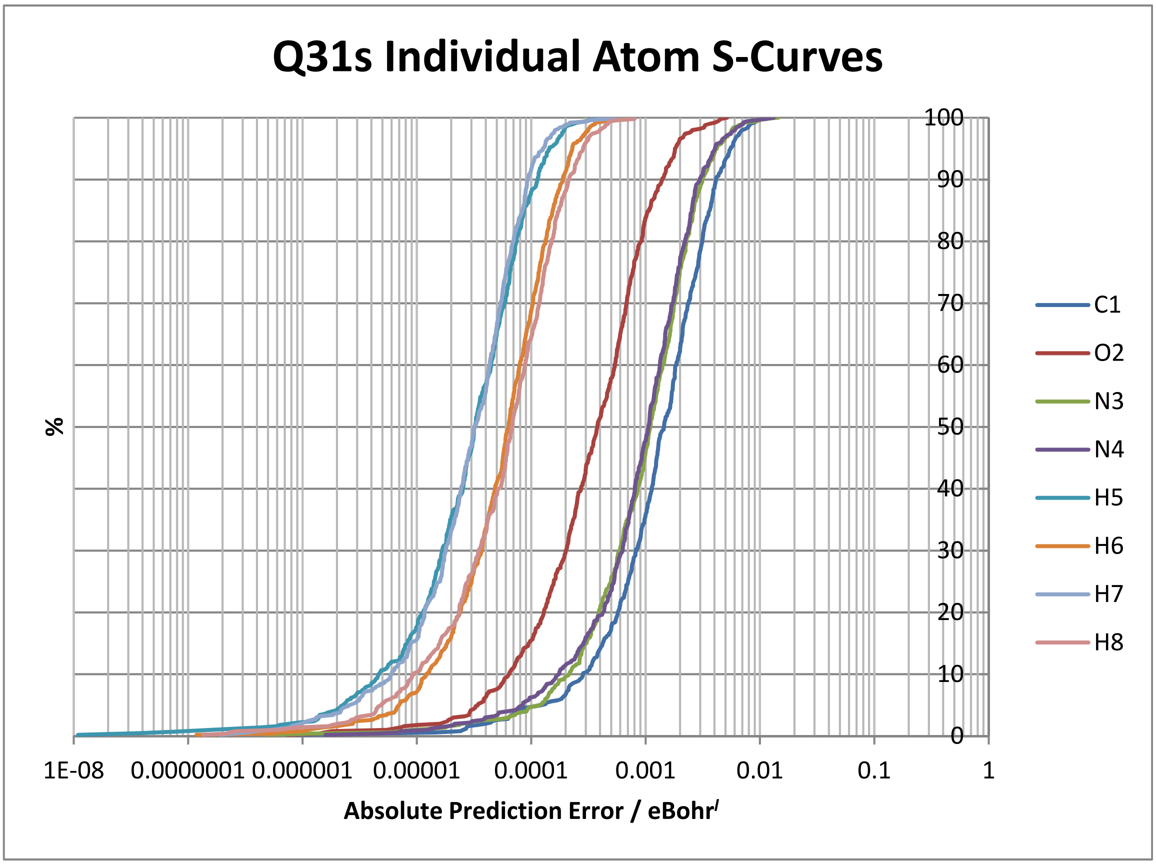

Figure S19. Q31s individual atom S-curves.

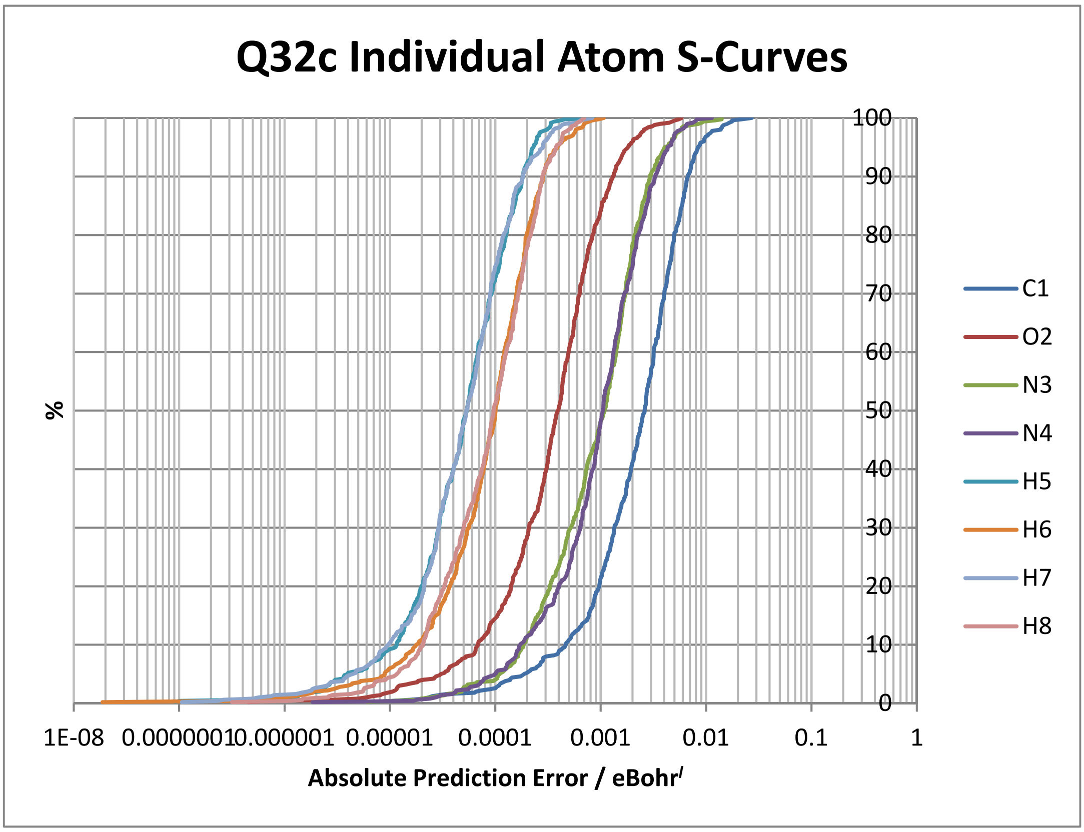

Figure S20. Q32c individual atom S-curves.

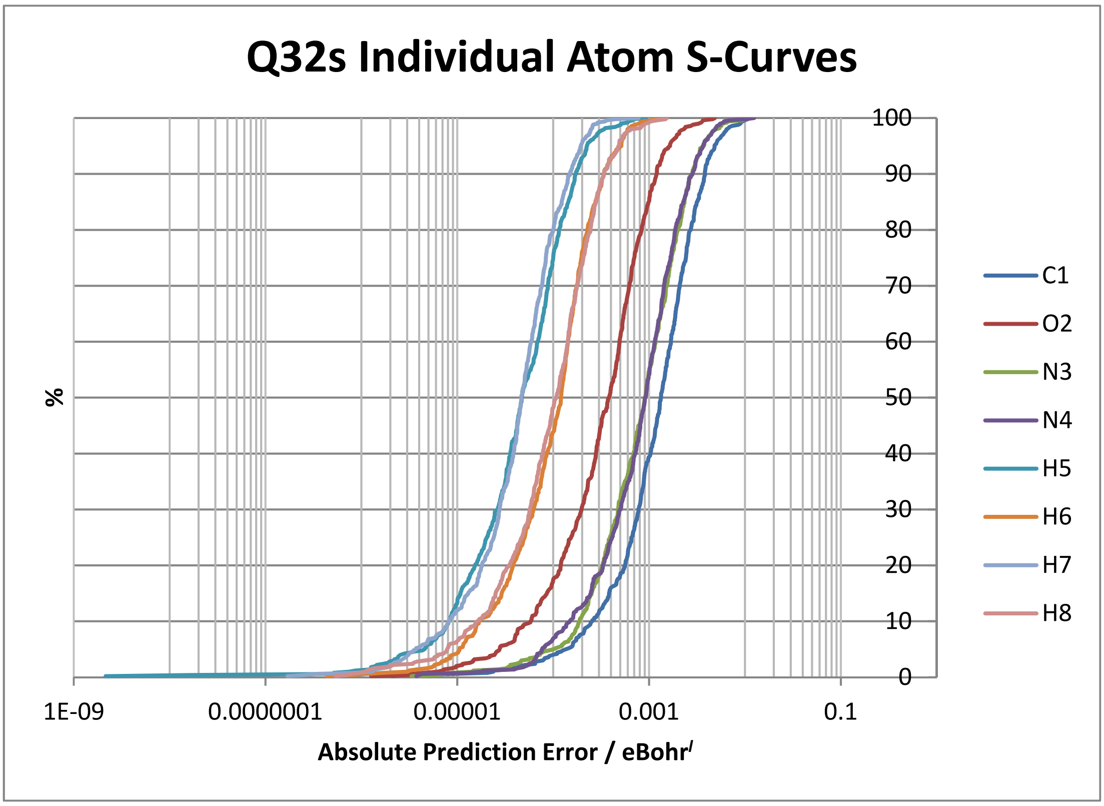

Figure S21. Q32s individual atom S-curves.

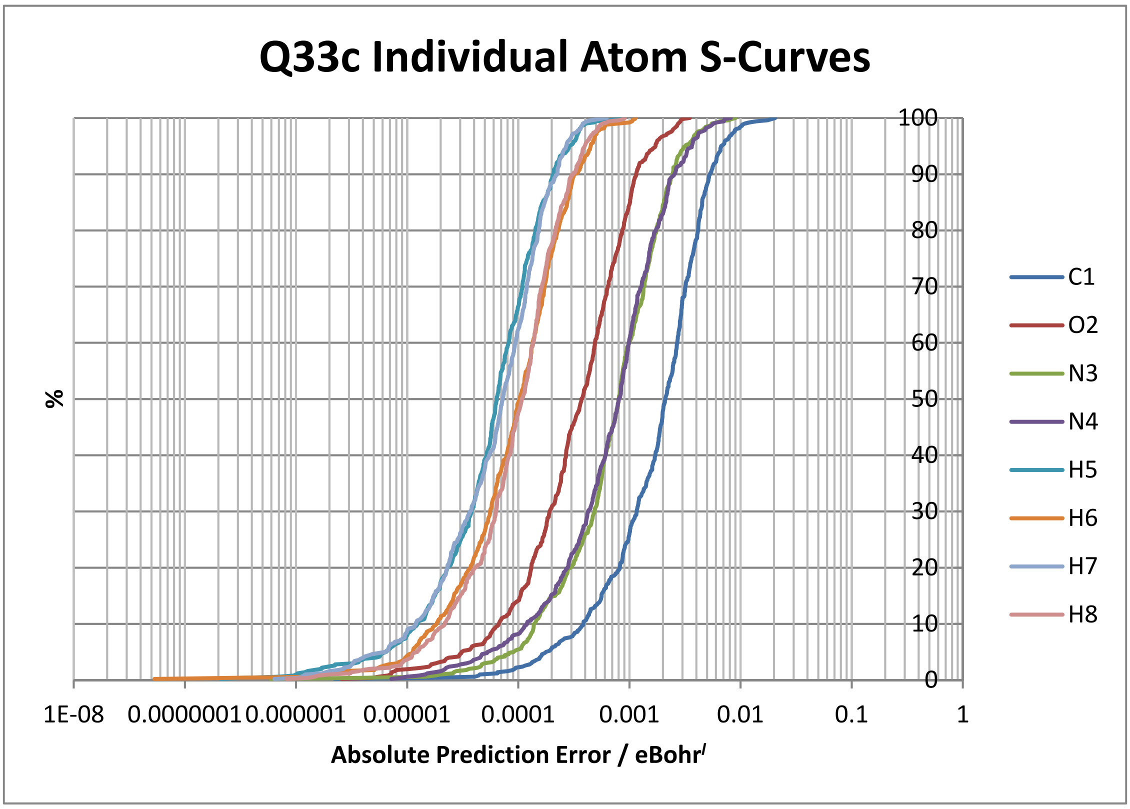

Figure S22. Q33c individual atom S-curves.

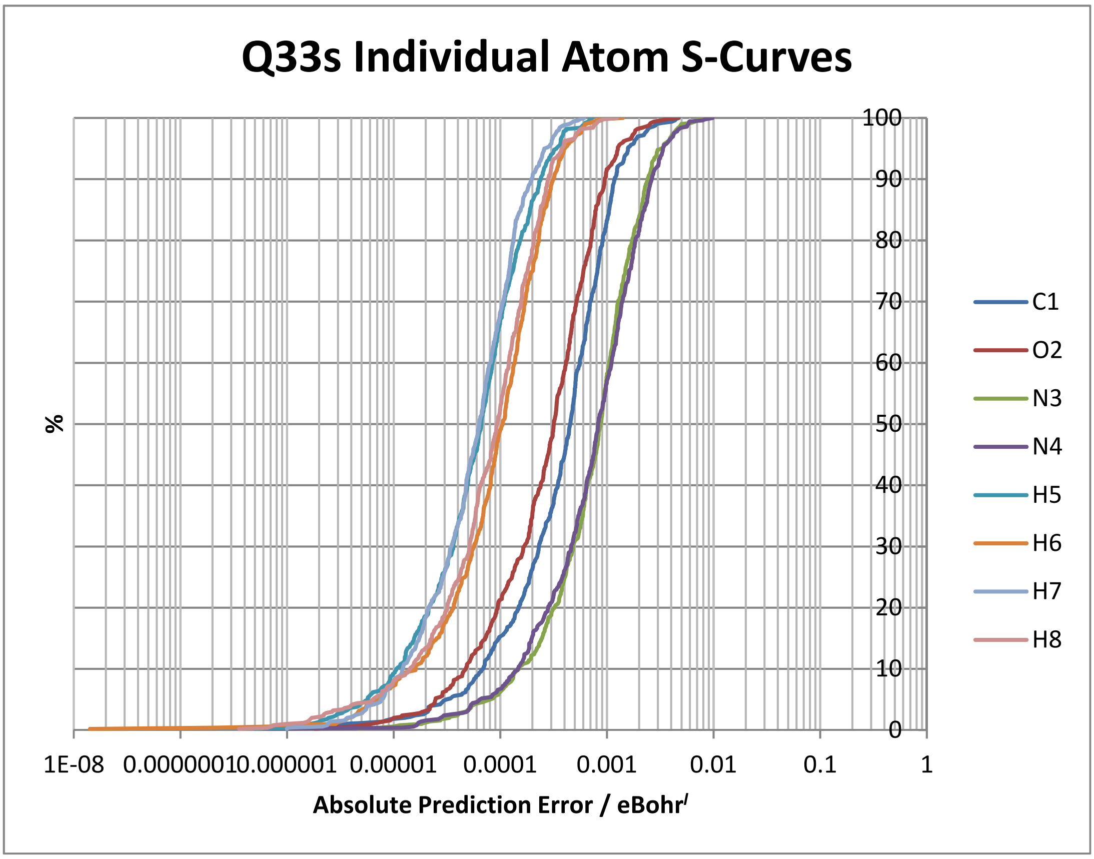

Figure S23. Q33s individual atom S-curves.

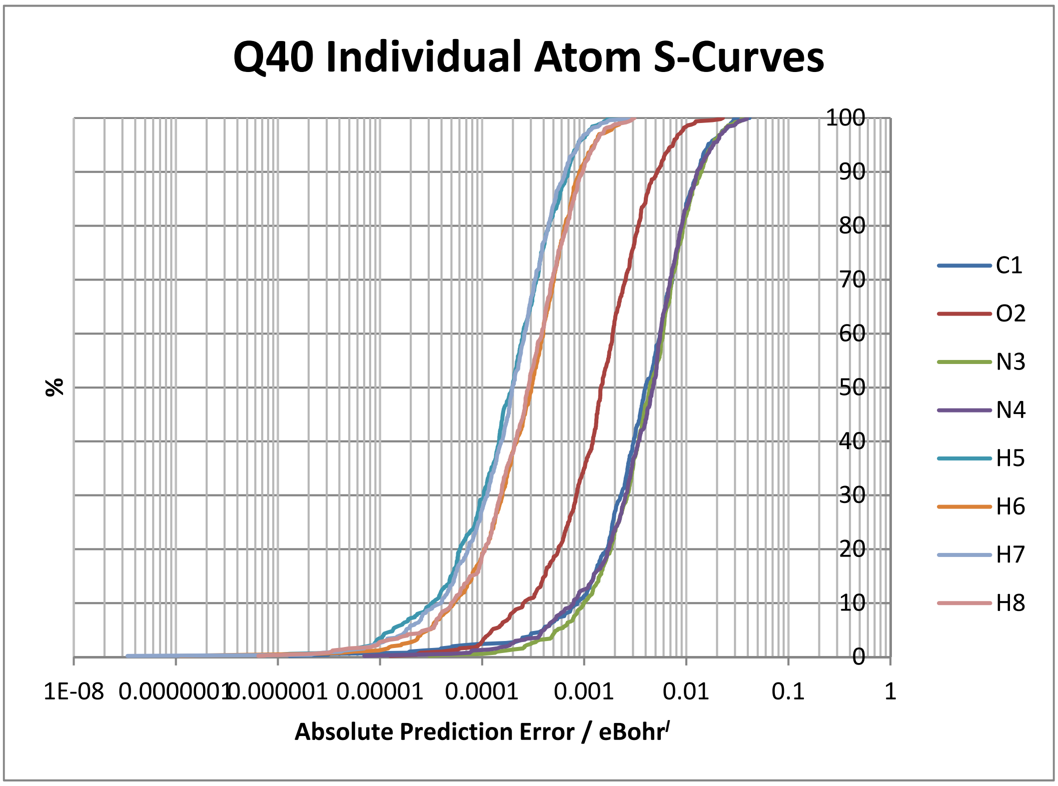

Figure S24. Q40 individual atom S-curves.

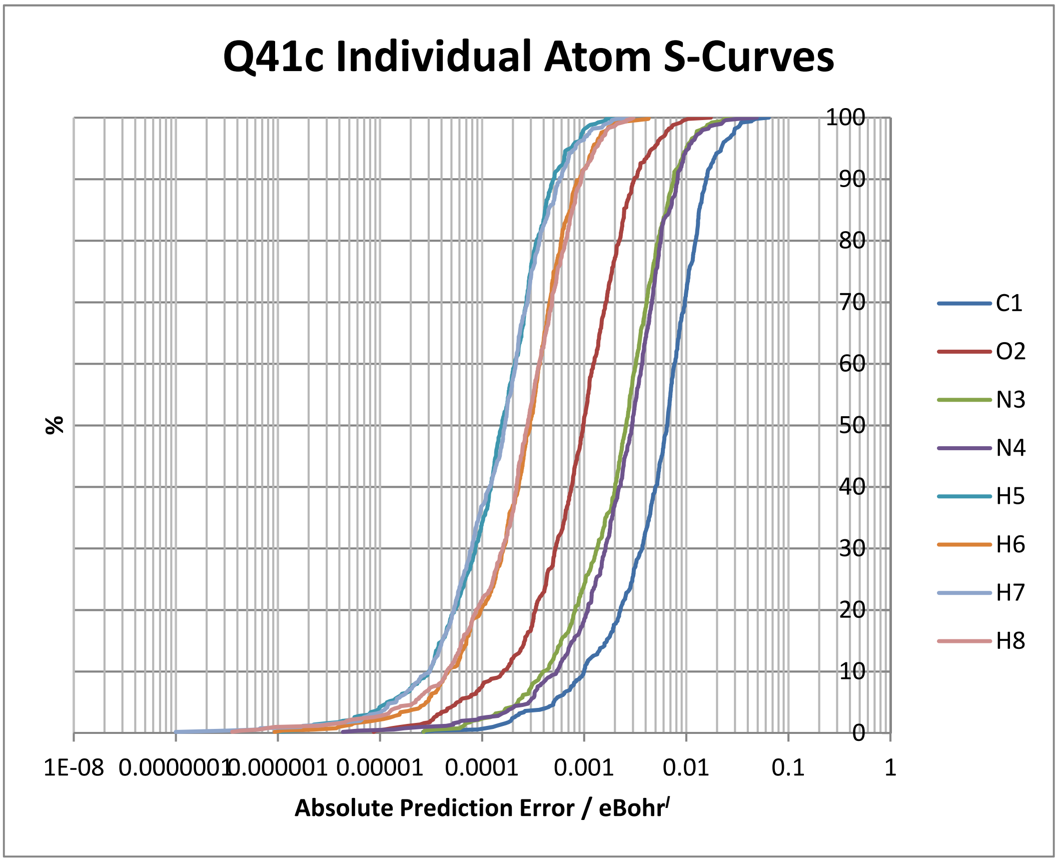

Figure S25. Q41c individual atom S-curves.

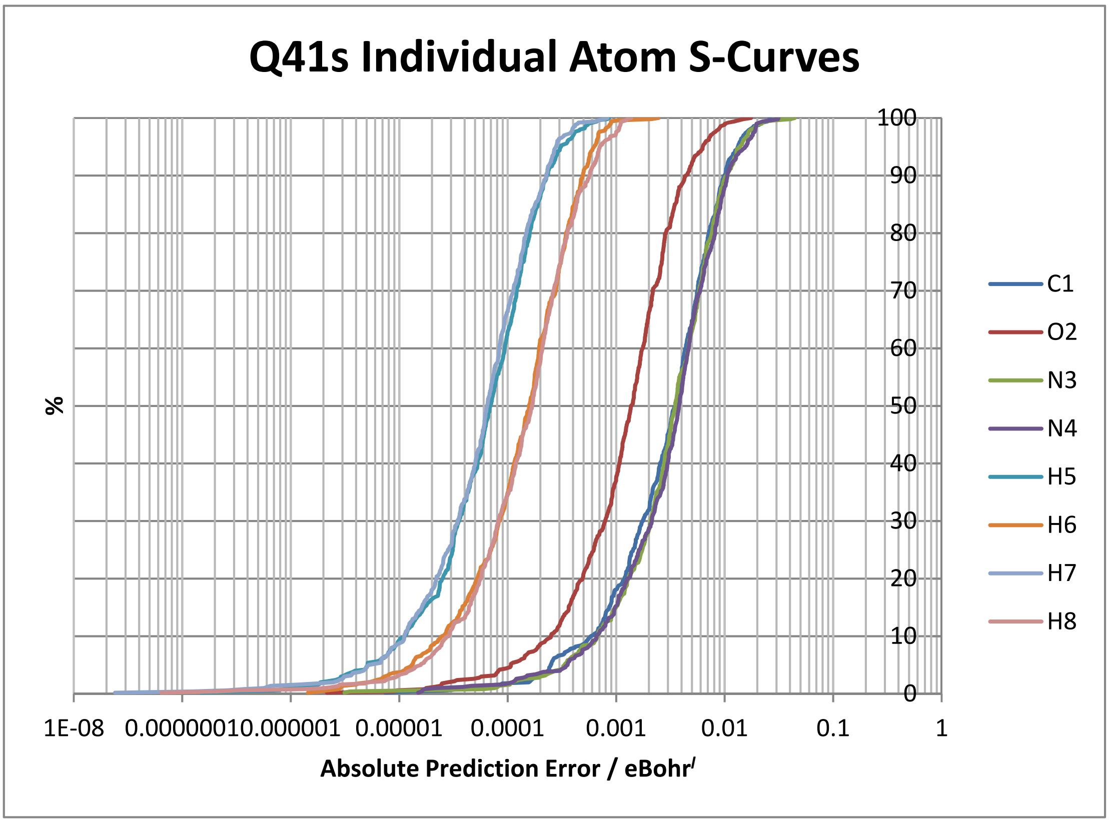

Figure S26. Q41s individual atom S-curves.

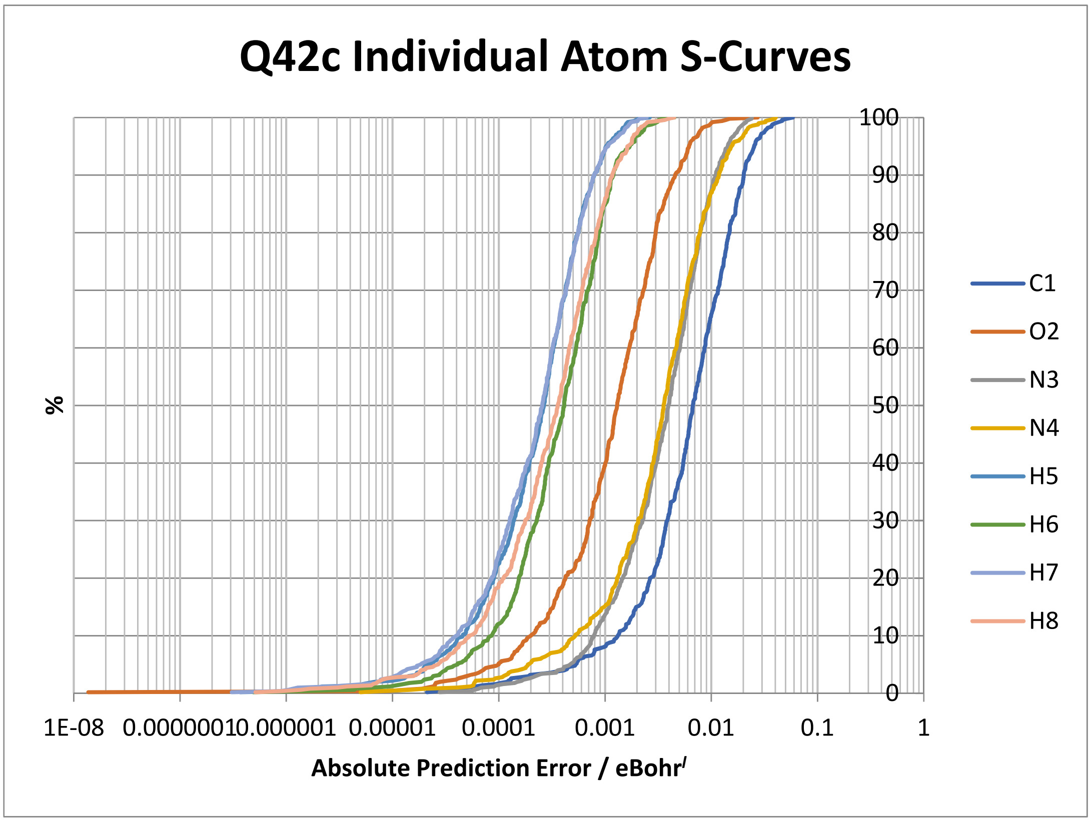

Figure S27. Q42c individual atom S-curves.

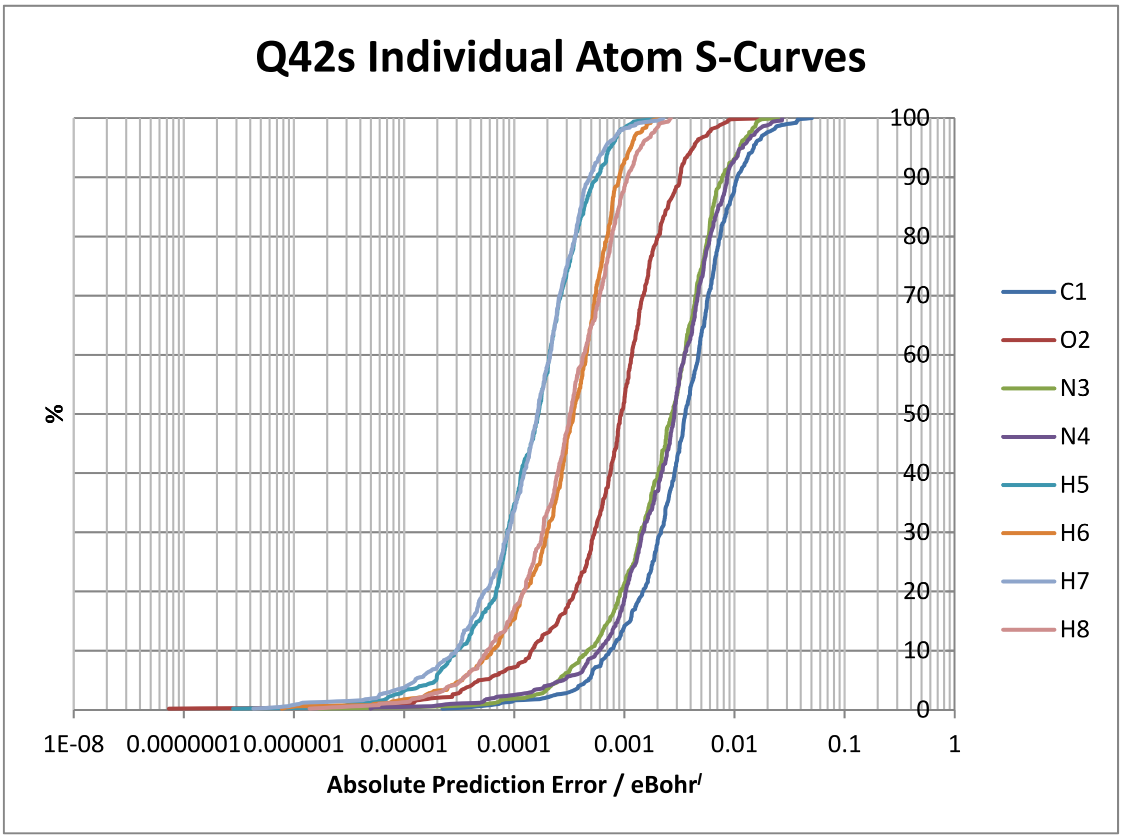

Figure S28. Q42s individual atom S-curves.

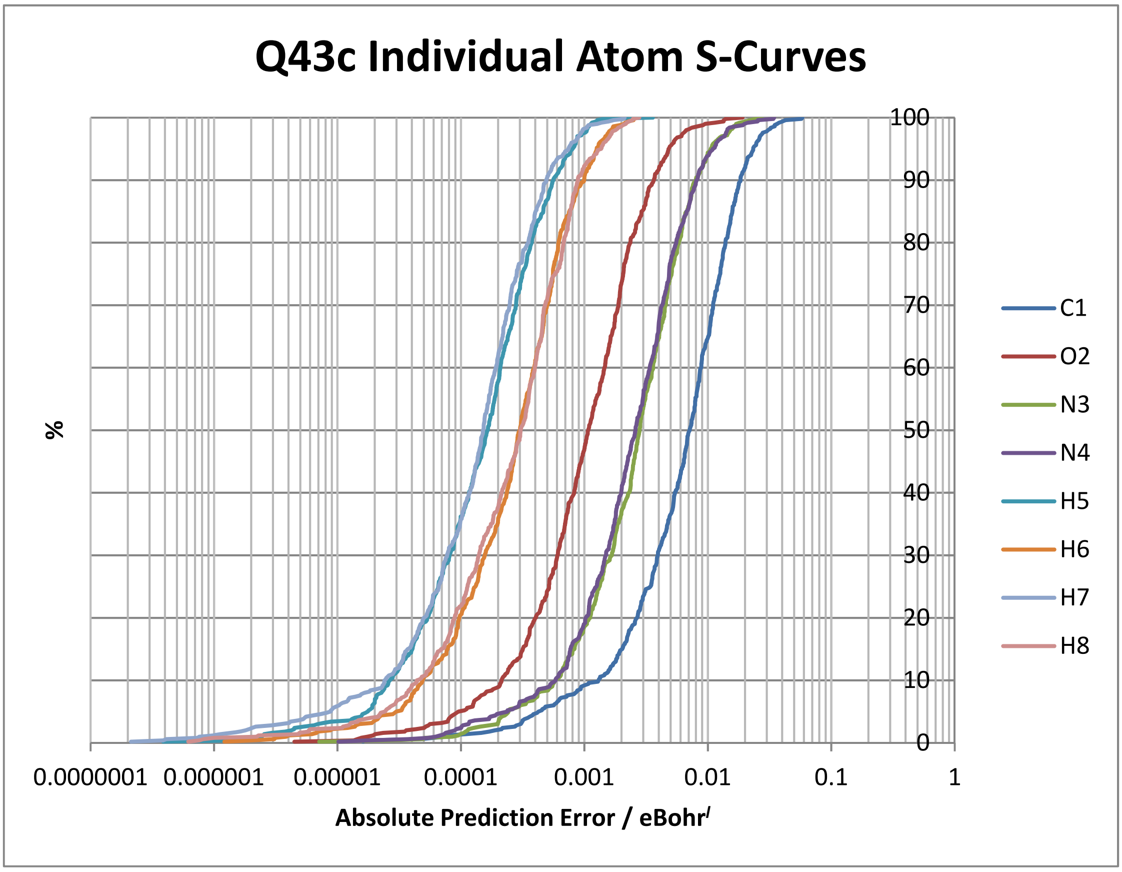

Figure S29. Q43c individual atom S-curves.

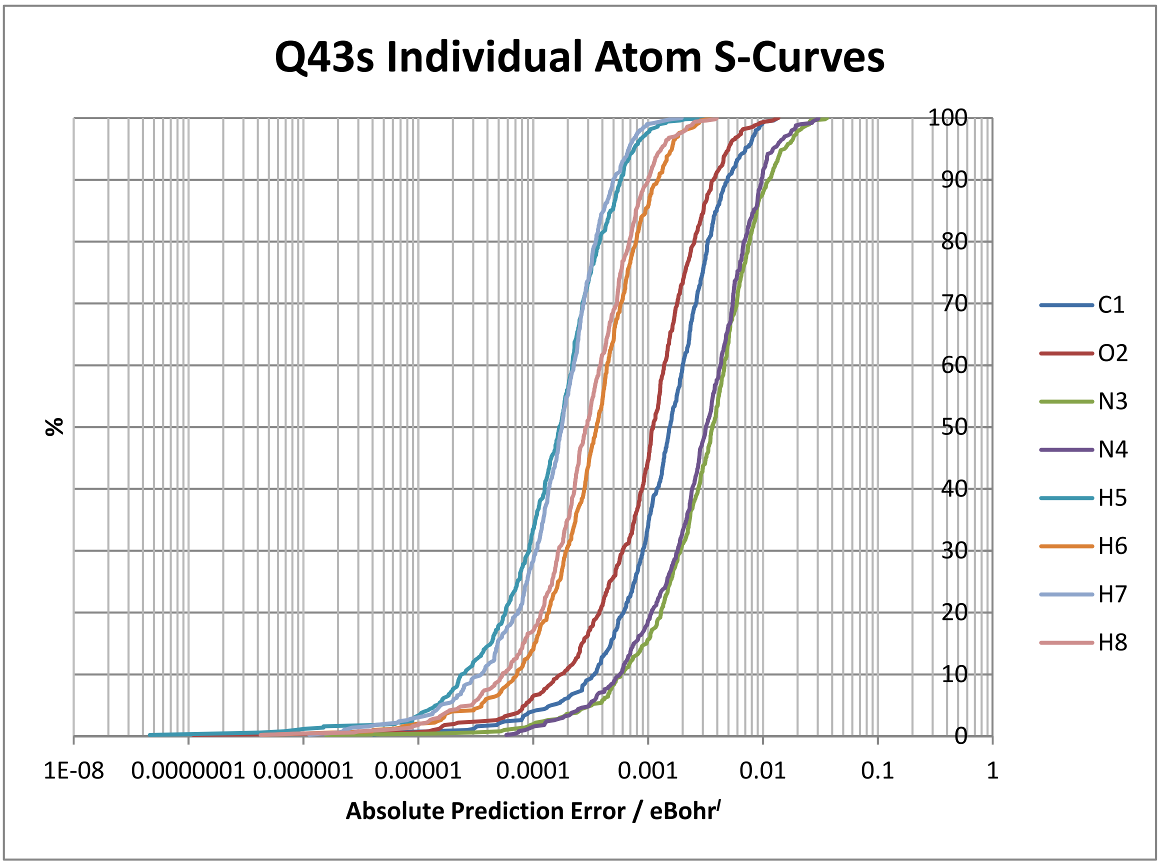

Figure S30. Q43s individual atom S-curves.

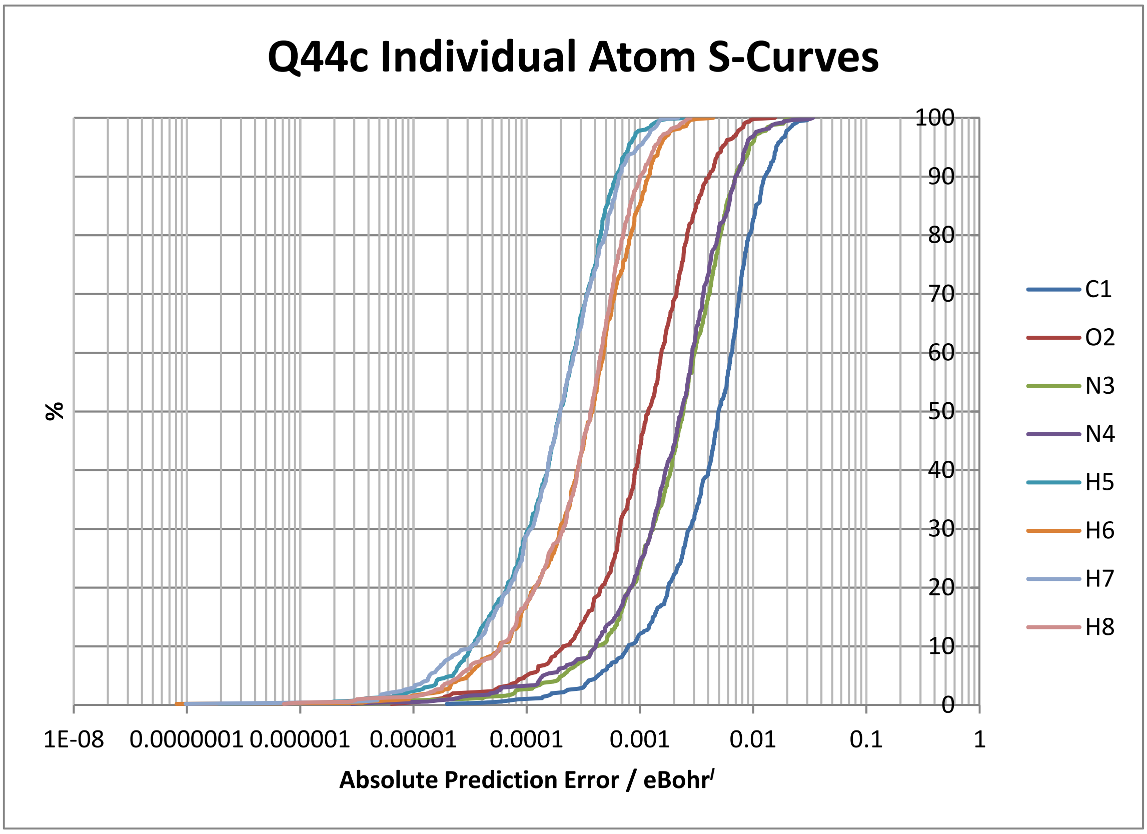

Figure S31. Q44c individual atom S-curves.

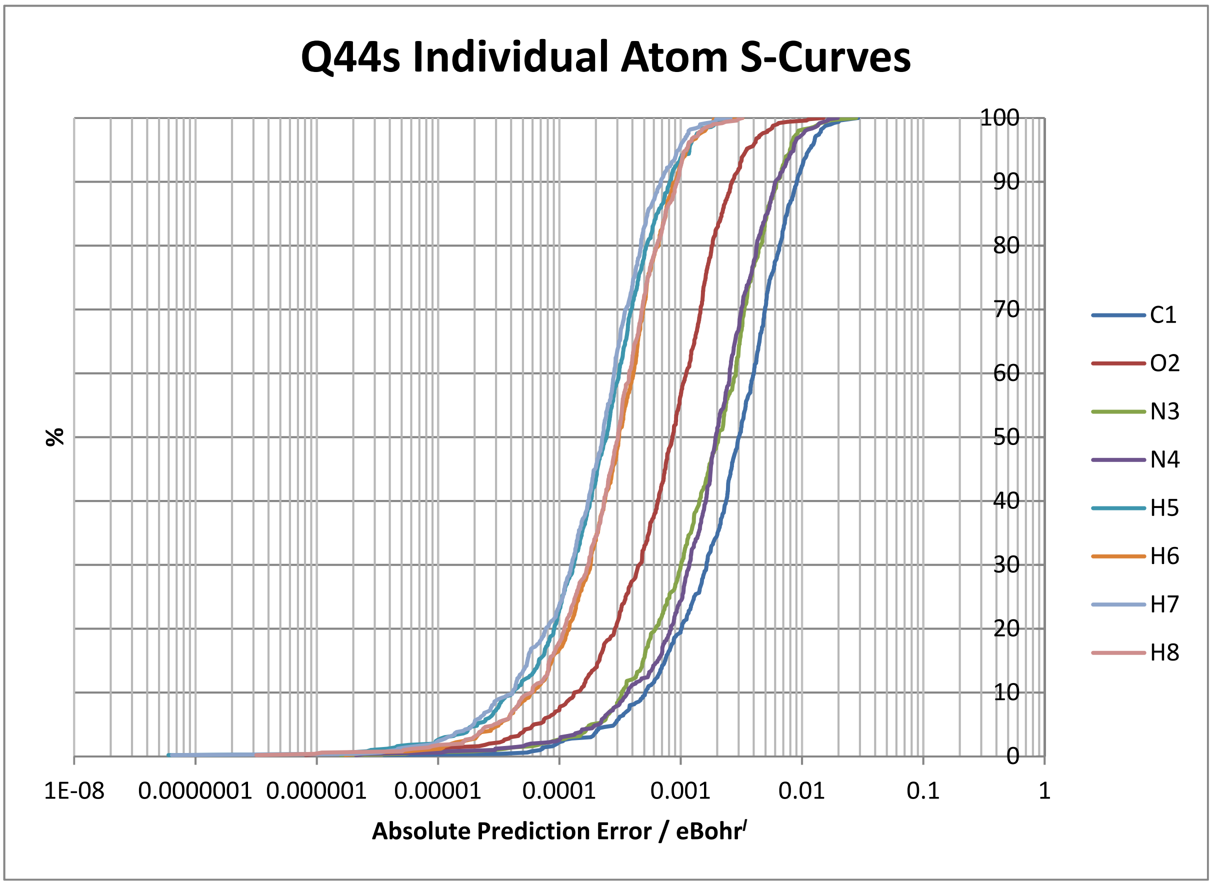

Figure S32. Q44s individual atom S-curves.

3. ADHAG non-bonded parameters

Table S7. ADHAG non-bonded parameter values for the 12-6 potential.

Atom	A / Å^{12} kJ mol^{-1}	B / Å^{6} kJ mol^{-1}
**C**	13,534,048	12,606.560
**O**	3,440,600	3,900.368
**N**	10,891,864	10,891,864

The above values are the parameters derived in previous work[2,3] and used in the dimer optimisations covered in Section 3.2.2 and 3.2.3 of this work. They are for the 12-6 non-bonded potential of the form:

$$U(r_{ij}) = \frac{A_{ij}}{r_{ij}^{12}} - \frac{B_{ij}}{r_{ij}^{6}} \qquad (S1)$$

with the following mixing rules:

$$A_{ij} = \sqrt{A_i A_j} \ \ and \ \ B_{ij} = \sqrt{B_i B_j} \qquad (S2)$$

4. FFLUX simulation RMSD

The following 5 figures (one for each *Dn* (n=1,2,3,4,5) urea dimer) are the RMSD plots of simulated urea dimers against the equivalent B3LYP/aug-cc-pVTZ (GAUSSIAN) optimised dimer, with all atoms included. The lowest L' value that is sufficient (indicated by convergent rather than divergent curves) to preserve the structure of the B3LYP equivalent is $L' = 2$ with the exception of dimer *D3* where $L' = 1$ is sufficient. The average RMSD values over the last 1,000 steps ($\overline{RMSD}$) are given in the figure legend.

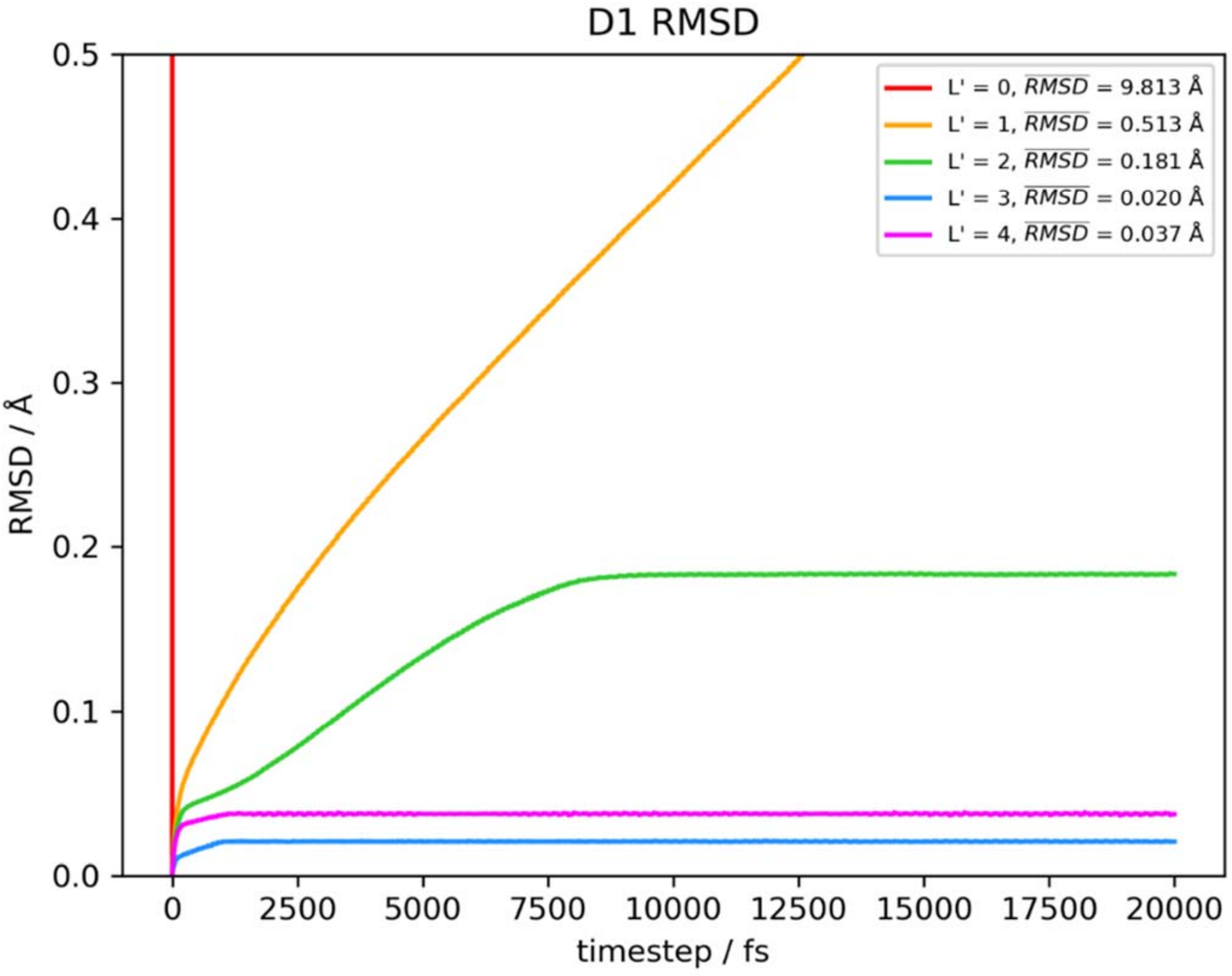

Figure S33. Dimer D1 FFLUX simulation RMSD *vs* its B3LYP optimised equivalent.

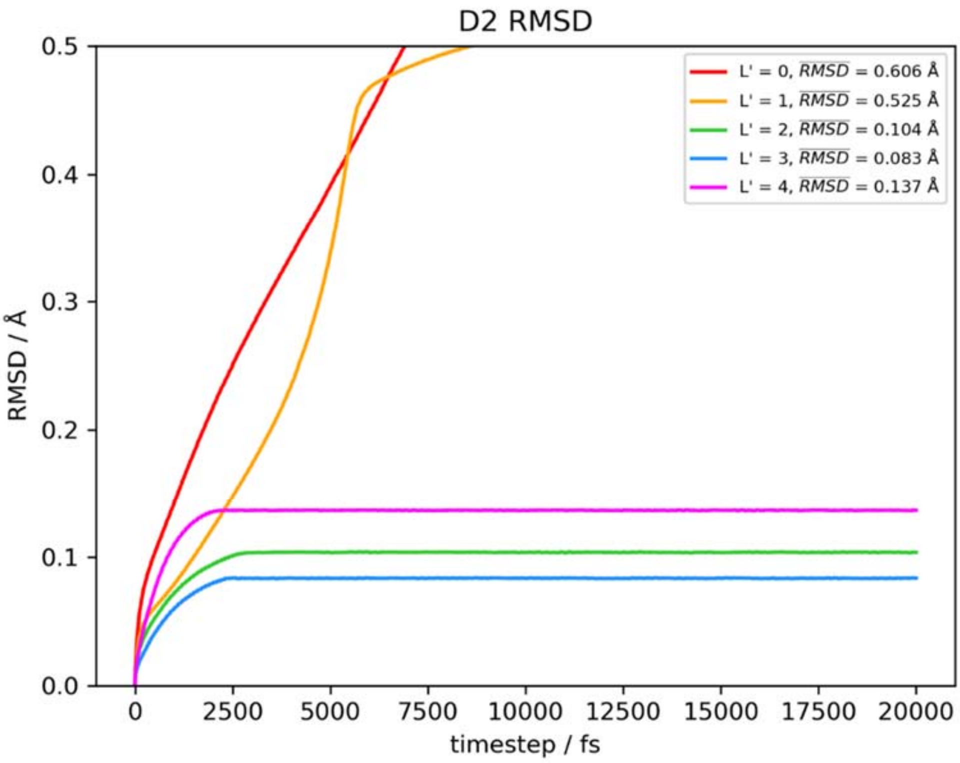

Figure S34. Dimer D2 FFLUX simulation RMSD *vs* its B3LYP optimised equivalent.

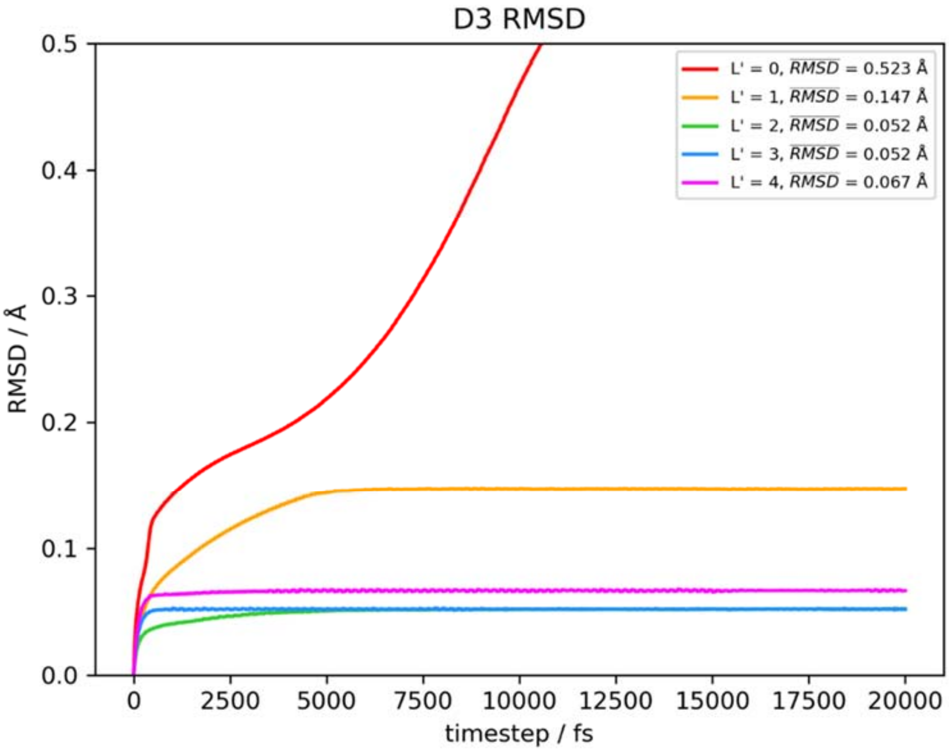

Figure S35. Dimer D3 FFLUX simulation RMSD *vs* its B3LYP optimised equivalent.

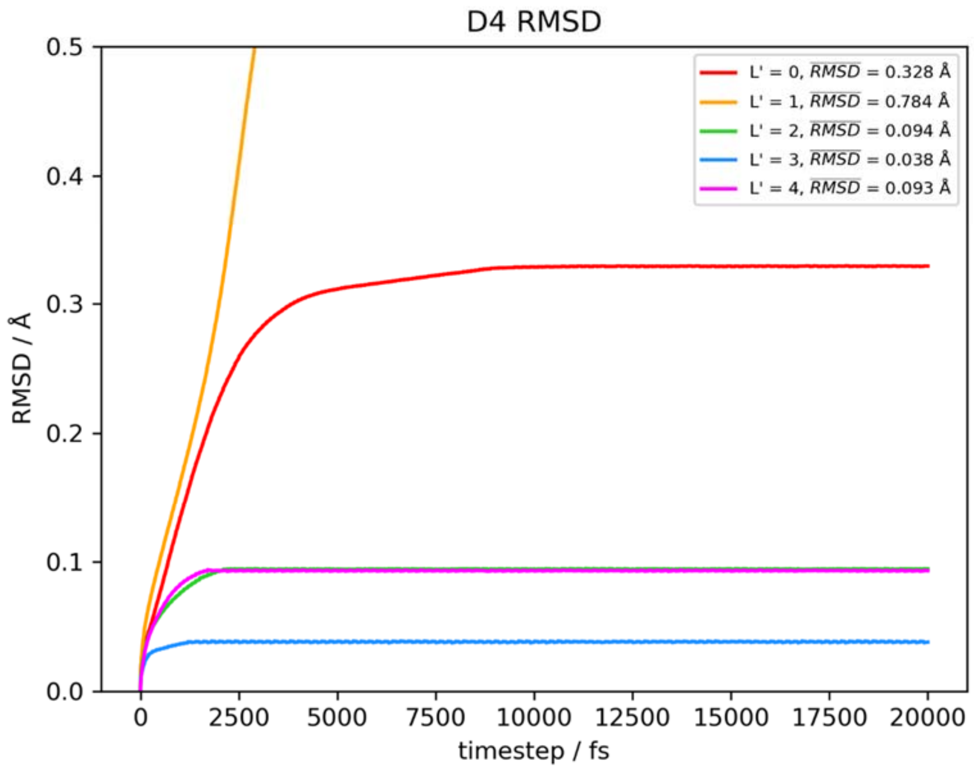

Figure S36. Dimer D4 FFLUX simulation RMSD *vs* its B3LYP optimised equivalent.

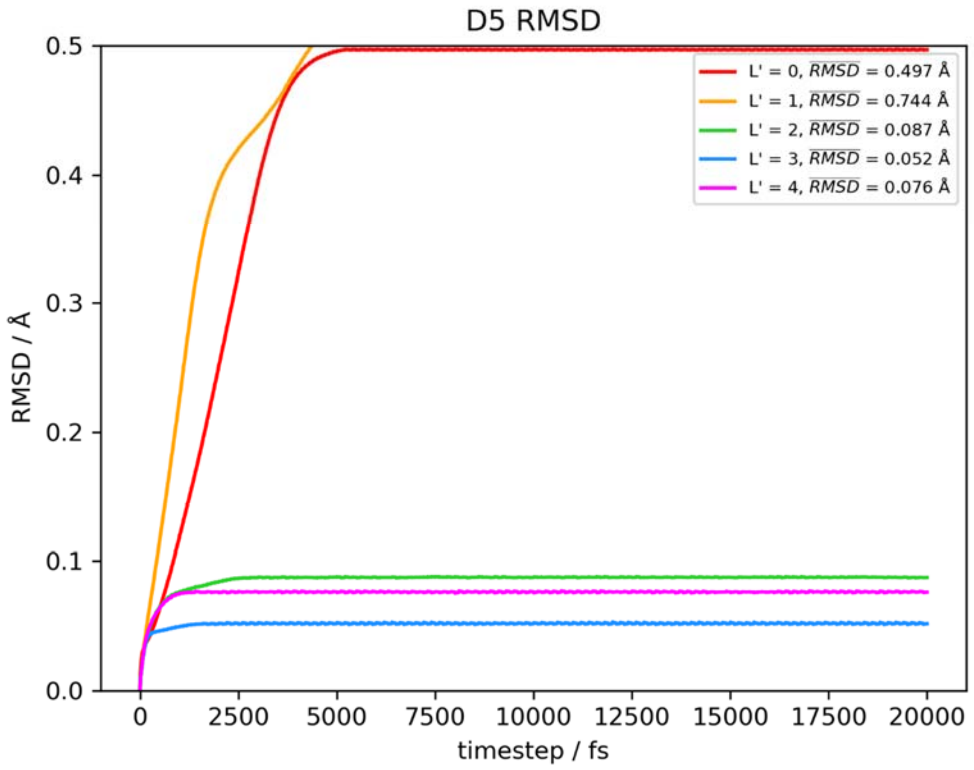

Figure S37. Dimer D5 FFLUX simulation RMSD *vs* its B3LYP optimised equivalent.

5. Training set mist plot

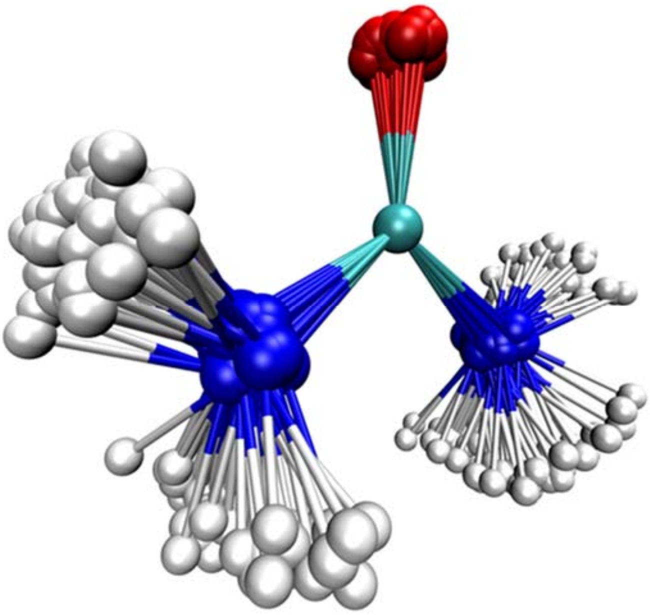

Figure S38. A visual representation of the input set of geometries used to train the GPR models.

6. Multipole moment tensor components for the FFLUX static moments run

The multipole tensor components of the B3LYP/aug-cc-pVTZ optimised monomer are given in the format just below. Their values are in the units of the program[4] DL_POLY_4s, *i.e.*, $proton\ charge \times \mathring{A}^{l}$, where l is the rank of multipole moment. The multipole moments are in the redundant, Cartesian form because such is required for the smooth particle mesh Ewald method used in DL_POLY_4.

Charge: q

Dipole: μ_x μ_y μ_z

Quadrupole: Θ_{xx} Θ_{xy} Θ_{xz} Θ_{yy} Θ_{yz} Θ_{zz}

Octupole: Ω_{xxx} Ω_{xxy} Ω_{xxz} Ω_{xyy} Ω_{xyz} Ω_{xzz} Ω_{yyy} Ω_{yyz} Ω_{yzz} Ω_{zzz}

Hexadecapole: Φ_{xxxx} Φ_{xxxy} Φ_{xxxz} Φ_{xxyy} Φ_{xxyz} Φ_{xxzz} Φ_{xyyy} Φ_{xyyz} Φ_{xyzz} Φ_{xzzz} Φ_{yyyy} Φ_{yyyz} Φ_{yyzz} Φ_{yzzz} Φ_{zzzz}

Table S8. C1 multipole moment tensor components.

1.949														
0.000	0.106	0.000												
0.250	0.000	0.000	-0.216	0.000	-0.479									
0.000	-0.027	0.000	0.000	0.000	0.000	0.068	0.000	0.025	0.000					
-0.078	0.000	0.000	-0.017	0.000	-0.057	0.000	0.000	0.000	0.000	-0.067	0.000	-0.048	0.000	-0.408

Table S9. O2 multipole moment tensor components.

-1.216														
0.000	0.207	0.000												
-1.437	0.000	0.000	-1.297	0.000	-1.442									
0.000	-0.067	0.000	0.000	0.000	0.000	-0.117	0.000	0.012	0.000					
-1.245	0.000	0.000	-0.334	0.000	-0.434	0.000	0.000	0.000	0.000	-0.789	0.000	-0.350	0.000	-1.377

Table S10. N3 multipole moment tensor components.

-1.187														
-0.030	-0.010	0.000												
-1.232	-0.011	0.000	-1.189	0.000	-1.626									
0.089	0.093	0.000	0.025	0.000	0.052	-0.010	0.000	0.048	0.000					
-0.732	-0.046	0.000	-0.255	0.000	-0.446	0.025	0.000	0.014	0.000	-0.661	0.000	-0.455	0.000	-1.878

Table S11. N4 multipole moment tensor components.

-1.187														
0.030	-0.010	0.000												
-1.232	0.011	0.000	-1.189	0.000	-1.626									
-0.089	0.093	0.000	-0.025	0.000	-0.052	-0.010	0.000	0.048	0.000					
-0.732	0.046	0.000	-0.255	0.000	-0.446	-0.025	0.000	-0.014	0.000	-0.661	0.000	-0.455	0.000	-1.878

Table S12. H5 multipole moment tensor components.

0.425														
-0.080	-0.043	0.000												
-0.067	0.004	0.000	-0.069	0.000	-0.074									
-0.049	-0.002	0.000	-0.017	0.000	-0.024	-0.030	0.000	-0.013	0.000					
-0.056	0.000	0.000	-0.016	0.000	-0.020	-0.005	0.000	-0.003	0.000	-0.041	0.000	-0.015	0.000	-0.044

Table S13. H6 multipole moment tensor components.

0.396														
-0.003	0.095	0.000												
-0.078	0.001	0.000	-0.068	0.000	-0.081									
-0.007	0.026	0.000	-0.001	0.000	-0.002	0.053	0.000	0.031	0.000					
-0.043	0.005	0.000	-0.019	0.000	-0.016	0.002	0.000	0.001	0.000	-0.058	0.000	-0.025	0.000	-0.050

Table S14. H7 multipole moment tensor components.

0.425														
0.080	-0.043	0.000												
-0.067	-0.004	0.000	-0.069	0.000	-0.074									
0.049	-0.002	0.000	0.017	0.000	0.024	-0.030	0.000	-0.013	0.000					
-0.056	0.000	0.000	-0.016	0.000	-0.020	0.005	0.000	0.003	0.000	-0.041	0.000	-0.015	0.000	-0.044

Table S15. H8 multipole moment tensor components.

0.396														
0.003	0.095	0.000												
-0.078	-0.001	0.000	-0.068	0.000	-0.081									
0.007	0.026	0.000	0.001	0.000	0.002	0.053	0.000	0.031	0.000					
-0.043	-0.005	0.000	-0.019	0.000	-0.016	-0.002	0.000	-0.001	0.000	-0.058	0.000	-0.025	0.000	-0.050

7. References

(1) Frisch, M. J.; Trucks, G. W.; Schlegel, H. B.; Scuseria, G. E.; Robb, M. A.; Cheeseman, J. R.; Scalmani, G.; Barone, V.; Mennucci, B.; Petersson, G. A. *GAUSSIAN09, revision D.01*; Gaussian Inc.: Wallingford, CT, USA, 2013.

(2) Hagler, A.; Huler, E.; Lifson, S. Energy Functions for Peptides and Proteins. I. Derivation of a Consistent Force Field Including the Hydrogen Bond from Amide Crystals. *J.Am.Chem.Soc.* **1974**, *96*, 5319-5327.

(3) Brown, M. L.; Skelton, J. M.; Popelier, P. L. A. Construction of a Gaussian Process Regression Model of Formamide for Use in Molecular Simulations. *J.Phys.Chem.A* **2023**, *127*, 1702-1714.

(4) *The DL_POLY_4 User Manual, CCLRC Daresbury Laboratory, Warrington, Great Britain*; CCLRC Daresbury Laboratory: Warrington, Great Britain, 2018.
